# Supplementary material for: Probing sub-5 Ångstrom micropores in carbon for precise light olefin/paraffin separation
Source: Nat Commun. 2023 Mar 2;14:1197. doi: 10.1038/s41467-023-36890-6 (PMC9981619; doi:10.1038/s41467-023-36890-6)
Supplement: Supplementary file 1 — Supplementary Information [file 41467_2023_36890_MOESM1_ESM.pdf]

Supplementary Information

Probing sub-5 Ångstrom micropores in carbon for precise light olefins/paraffins separation

Shengjun Du, Jiawu Huang, Matthew R. Ryder, Luke L. Daemen, Cuiting Yang, Hongjun Zhang, Panchao Yin, Yuyan Lai, Jing Xiao,* Sheng Dai,* Banglin Chen*

*Corresponding author. cejingxiao@scut.edu.cn; dais@ornl.gov; banglin.chen@fjnu.edu.cn

This PDF file includes:

Supplementary Methods

Supplementary Figures. 1 to 47

Supplementary Tables 1 to 11

Supplementary References

Supplementary Methods

Materials

Dopamine hydrochloride ($C_8H_{11}NO_2$•HCl, 98%) was purchased from Beijing Innochem Technology Co., Ltd. Anhydrous ethanol (C_2H_5OH, AR) and ammonia water (NH_4OH, 28–30%) were obtained from Tianjin Damao Chemical Reagent Factory. All reagents were used as received without further purification. Commercial activated carbon, named AC-1 (Specific surface area of 1800 m^2 g^{-1}; particle size of 5-8 mm) was purchased from Shanghai Aladdin Bio-Chem Technology Co., LTD (China). Commercial carbon molecular sieve, named CMS-1, was purchased from Kuraray Co., LTD (Japan). For adsorption tests, CO_2 (99.99%), N_2 (99.99%), H_2 (99.99%), He (99.999%) were purchased from Guangzhou Shengying Chemical Co., Ltd. Pure gases of C_2H_4 (99.9%), C_2H_6 (99.9%), C_3H_6 (99.9%), C_3H_8 (99.9%) and mixed gases of C_2H_4/C_2H_6 (50/50, v/v) and C_3H_6/C_3H_8 (50/50, v/v) were purchased from Air Liquide (China).

Characterization

The morphology of samples was analyzed by scanning electron microscope (SEM, Hitachi SU8220) at an accelerating voltage of 5.0 kV. The samples were deposited with a few nm thin gold layer via sputtering in order to increase the surface conductivity. The element distribution mappings in the samples were determined by high-resolution transmission electron (TEM) mapping (200 kV, ultrasonically dispersed in ethanol). The types of functional groups were recorded by Fourier transform infrared spectroscopy (FT-IR， Nicolet IS50-Nicolet Continuum) in the range of 500-4000 cm^{-1} with a KBr pellet technique. Raman spectrum was obtained using a HJY LabRAM Aramis with laser wavelength of 532 nm. The X-ray diffraction (XRD) analysis was conducted on Rigaku SmartLab 9 kW with a monochromatic Cu Kα radiation source (λ = 0.15406 nm). Data were collected at room temperature at 2θ=10°-80° and operating power of 40 kV. X-ray photoelectron spectroscopy (XPS) was carried out on a Thermo Scientific K-Alpha. The Avantage software was used to deconvolute the narrow-scan XPS spectra of the N 1s and O 1s of the samples. ^{13}C NMR analyses were carried out on a Bruker Avance III 500 MHz Wb. Thermal gravimetric analysis (TGA) was collected on a thermal analyzer (PE-Optima 8000) under the nitrogen atmosphere. The sample was heated from room temperature to 900 °C.

X-ray Scattering measurements

Small-angle X-ray Scattering (SAXS). SAXS experiments are performed at the instrument in beamlines (BL-16B1 and BL-19U) of Shanghai Synchrotron Radiation Facility (SSRF) with a Pilatus 2M detector. RAW software was used to convert the raw data to 1D results and the pore size distributions were calculated using the Irena package in Igor software.

Wide-Angle X-ray Scattering (WAXS). 2D raw WAXS data was obtained on Rigaku Homelab, equipped with FR-X rotating anode target X-ray source (about 70 mm sample-to-detector distance), and RAW software was used to convert the raw data to 1D results. The scattered photon intensity is evaluated as a function of the scattering vector q given by:

$$q = \frac{4\pi}{\lambda}\sin\theta = \frac{2\pi}{d} \quad (1)$$

where λ refers to the wavelength of the incident X-ray (Cu Kα, λ = 1.5418 Å), θ is half scattering angle between incoming and scattered radiation, and d is the characteristic real void size. Thus, d can be calculated according to a specific q at the detector.

Analysis of Mercury porosimetry

Mercury porosimetry analysis was performed using a Micromeritics AutoPore IV 9500 instrument. The equipment enables the measurement of pore sizes in the range of 0.003–350 μm. The temperature of the test environment was 298 K. The samples were outgassed to remove any other species that were physically adsorbed on the samples. The mercury pressure ranged from 0.5 psi to 33000 psi.

Isosteric heat of adsorption

The isosteric heat of adsorption can be estimated from the adsorption isotherms at three different temperatures 273 K, 286 K and 298 K by using the Virial equation:[1]

$$\ln P = \ln N + 1/T \sum_{i=0}^{m} a_i N_i + \sum_{j=0}^{n} b_j N_j \quad (2)$$

Here, P is the pressure (bar), N is the adsorption capacity (mmol g^{-1}), T is the temperature (K), a_i and b_j refer to Virial coefficients, and m, n represent the number of coefficients required to adequately describe the isotherms. The values of the Virial coefficients a_0 through a_m were then used to calculate the isosteric heat of absorption using the following expression:

$$Q_{st} = -R \sum_{i=0}^{m} a_i N_i \quad (3)$$

Where the Q_{st} is denoted as the coverage-dependent isosteric heat of adsorption, R refers to the ideal gas constant. The heat enthalpy of C_2H_4 and C_3H_6 are determined using the isotherms data in the pressure range from 0-1.0 bar (at 273 to 298 K).

Supplementary Figures. 1-47

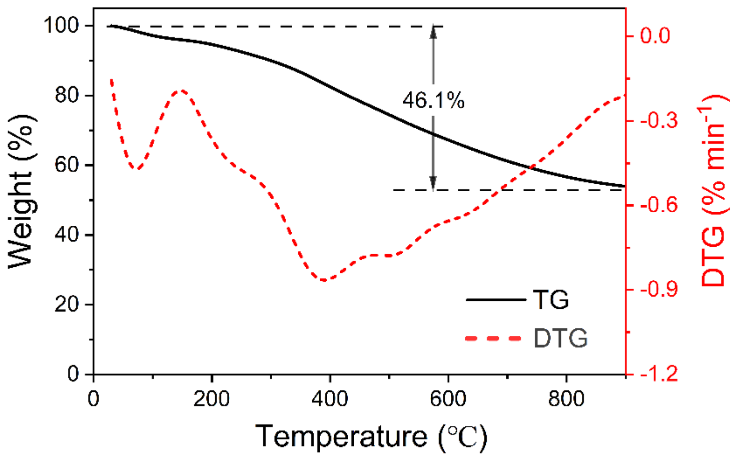

Supplementary Figure 1. TG and DTG curves of PDA polymer.

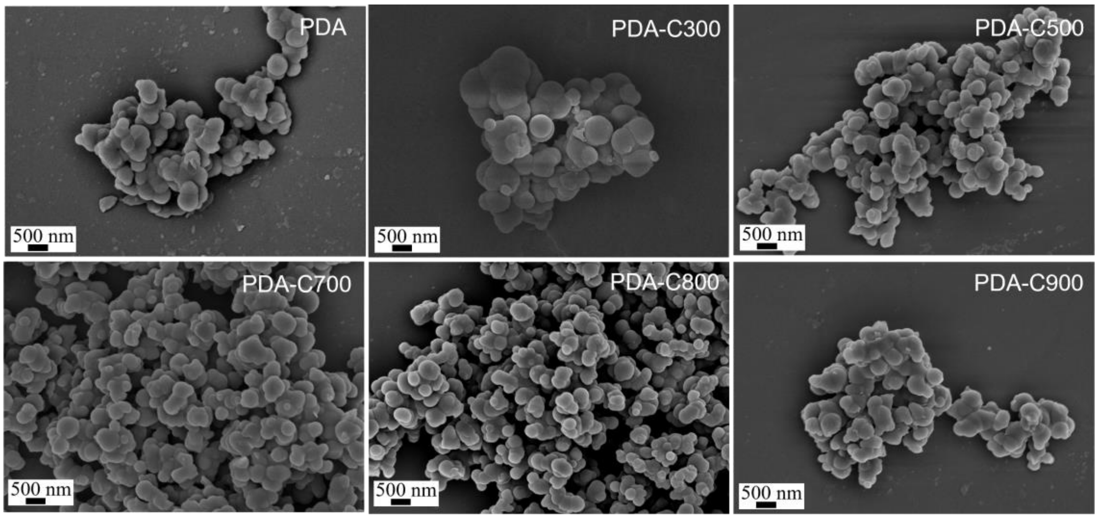

Supplementary Figure 2. SEM images of PDA and PDA-Cx.

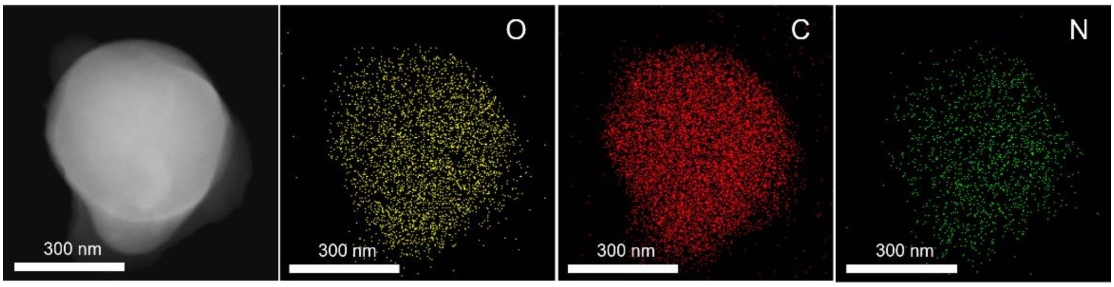

Supplementary Figure 3. Elemental mappings of oxygen, carbon and nitrogen on PDA based on TEM images.

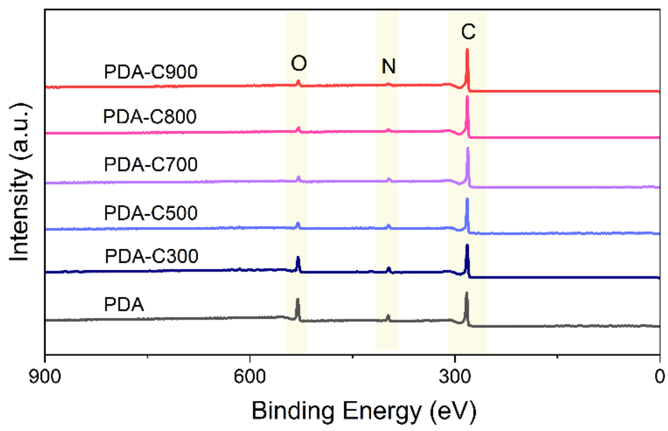

Supplementary Figure 4. XPS survey spectra of PDA and PDA-Cx.

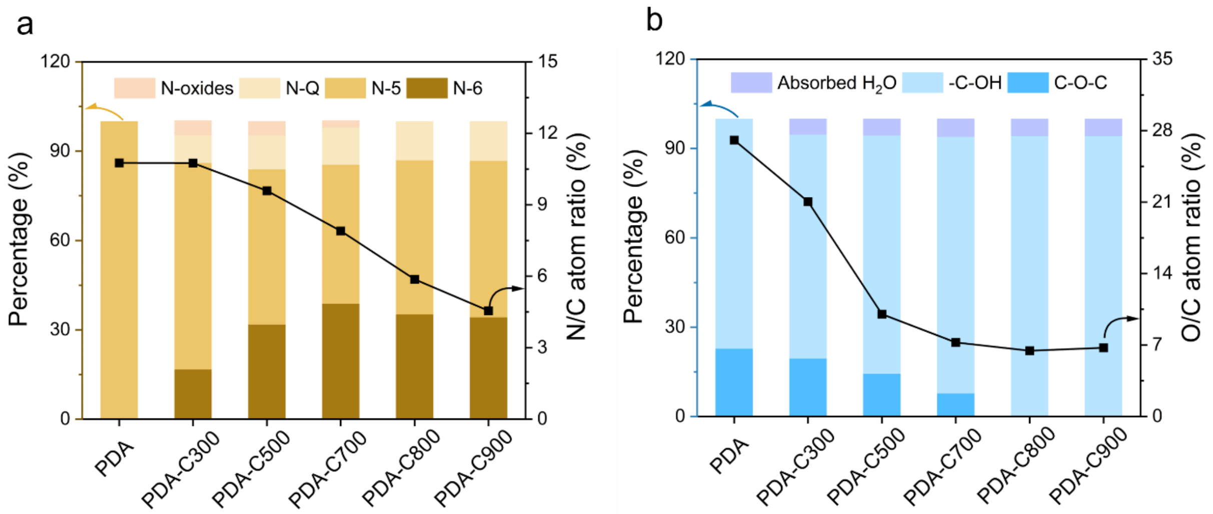

Supplementary Figure 5. Relative percentages of different types of (a) N and N/C atom ratio and (b) O and O/C atom ratio on PDA and PDA-Cx based on the XPS analysis.

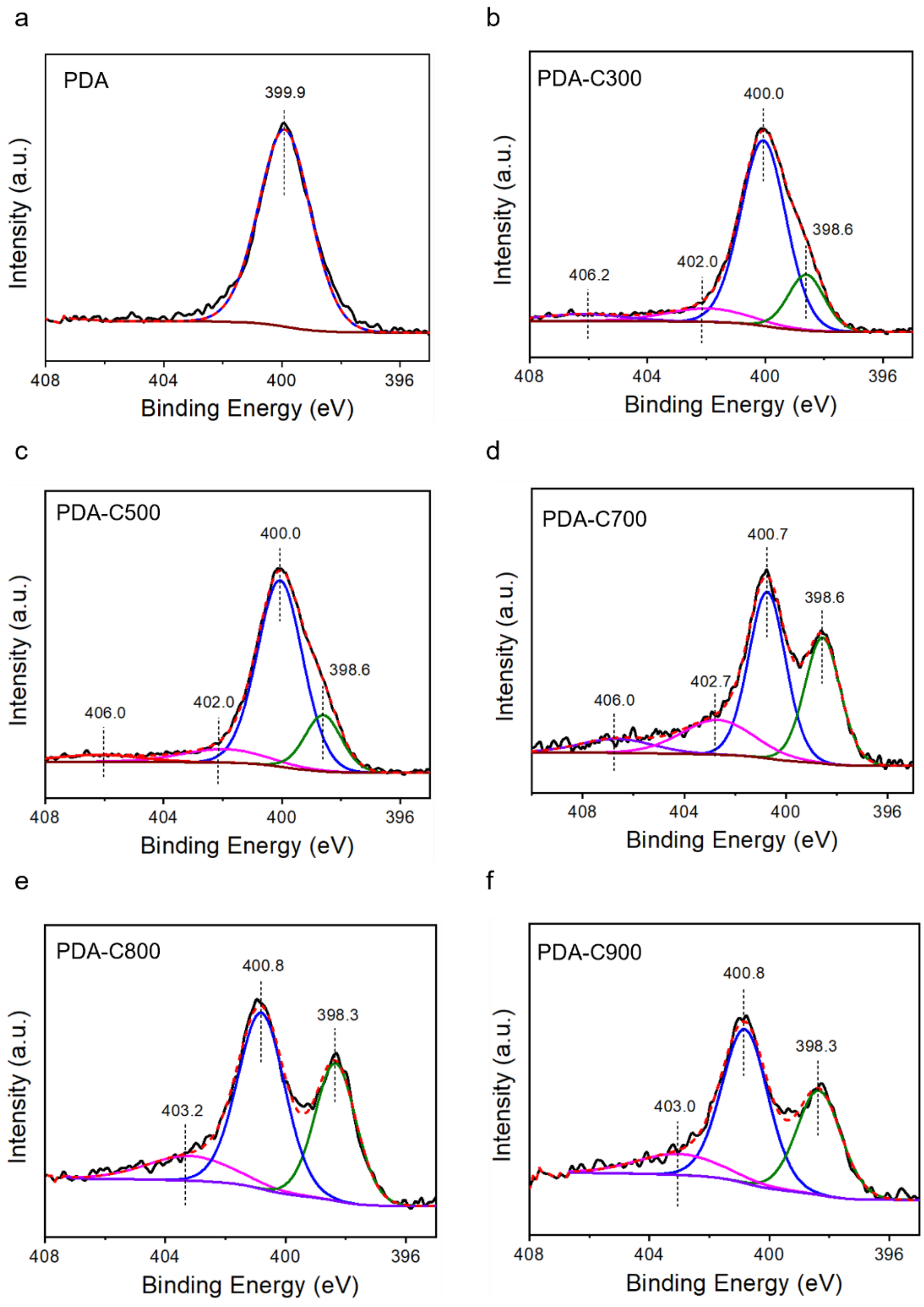

Supplementary Figure 6. High resolution XPS spectra of N1s for PDA and PDA-Cx.

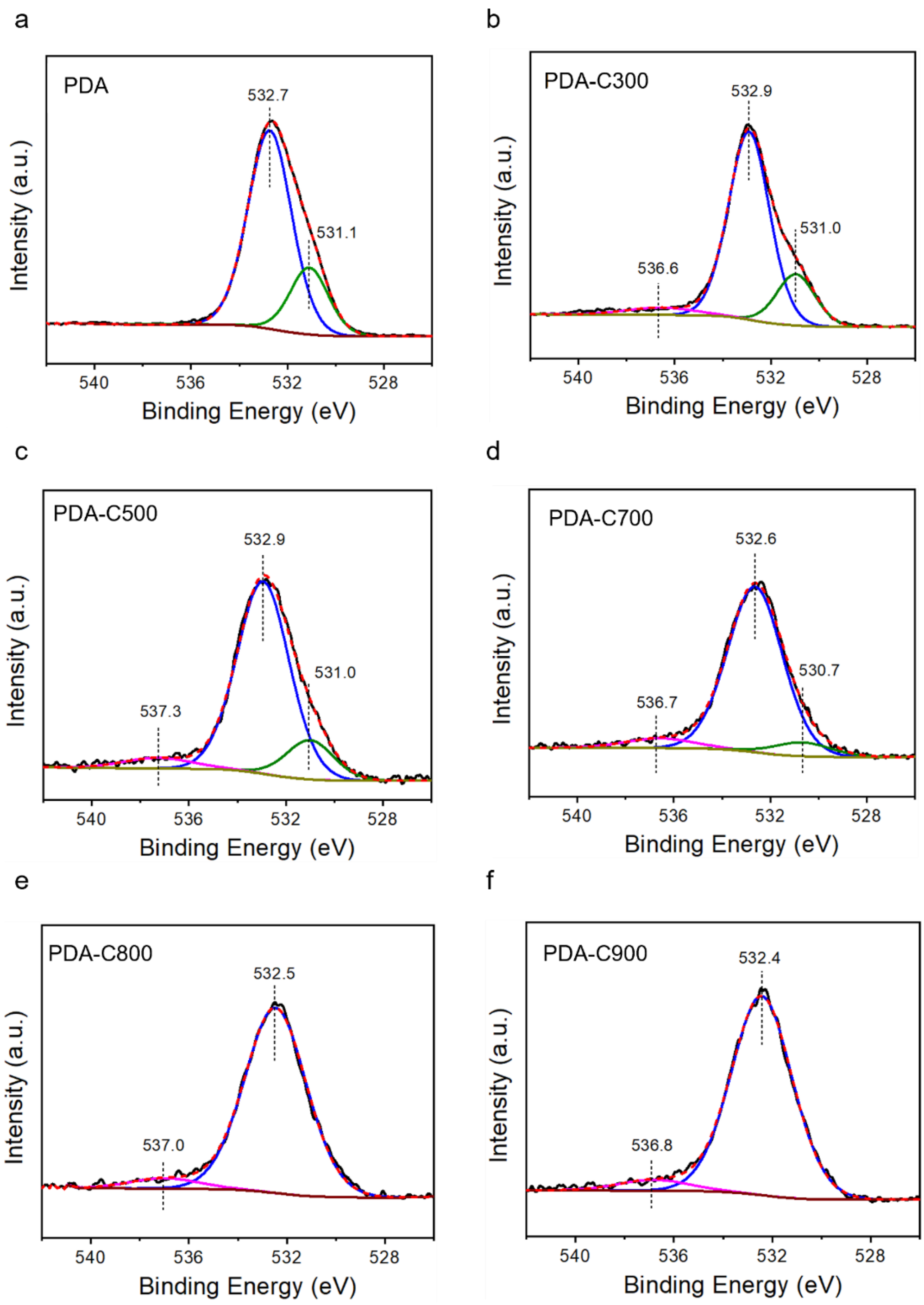

Supplementary Figure 7. High resolution XPS spectra of O1s for PDA and PDA-Cx.

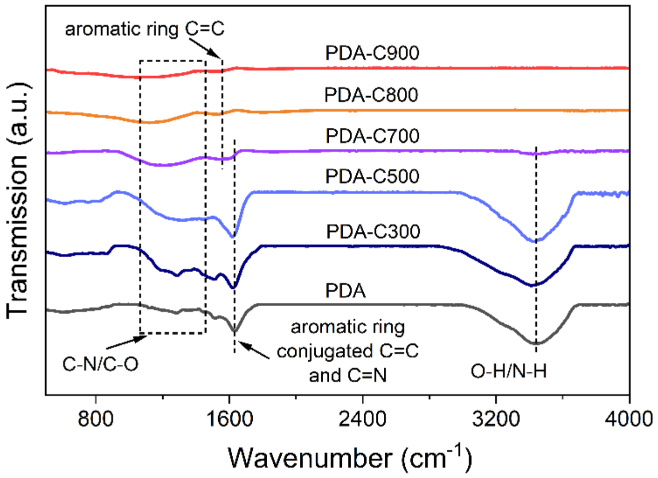

Supplementary Figure 8. FT-IR spectra of PDA and PDA-Cx (The reduction in peak intensities of heteroatom functional groups from PDA to PDA-C900 suggests that the cleavage bond occurred in the polymer backbone. The aromatic ring conjugated C=C and C=N groups at 1618 cm^{-1} were transferred to aromatic C=C groups at 1587 cm^{-1} with the increase in pyrolysis temperature, indicating the growth of graphene planes).

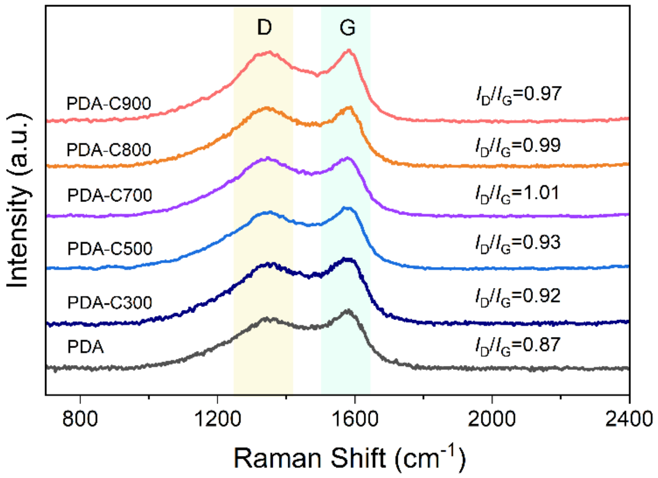

Supplementary Figure 9. Raman spectra of PDA and PDA-Cx.

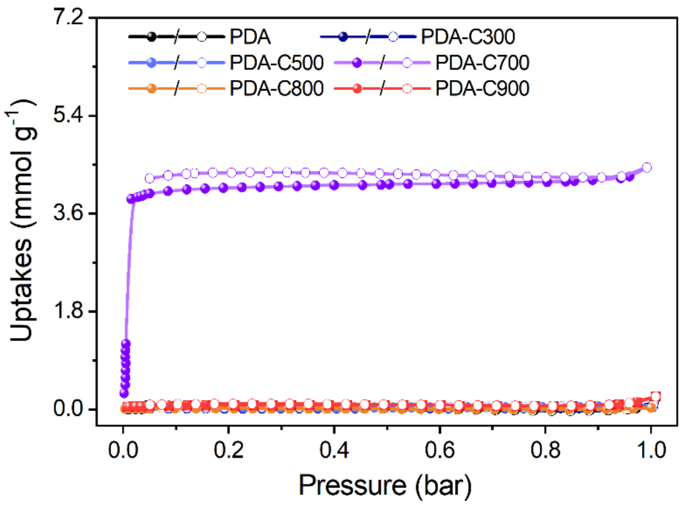

Supplementary Figure 10. N_2 adsorption (solid circles) and desorption (empty circles) isotherms at 77 K on PDA and PDA-Cx.

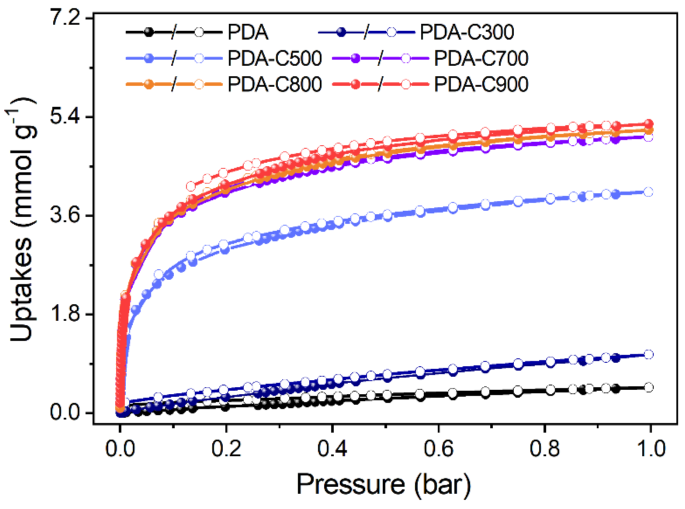

Supplementary Figure 11. H_2 adsorption (solid circles) and desorption (empty circles) isotherms of PDA and PDA-Cx at 77 K.

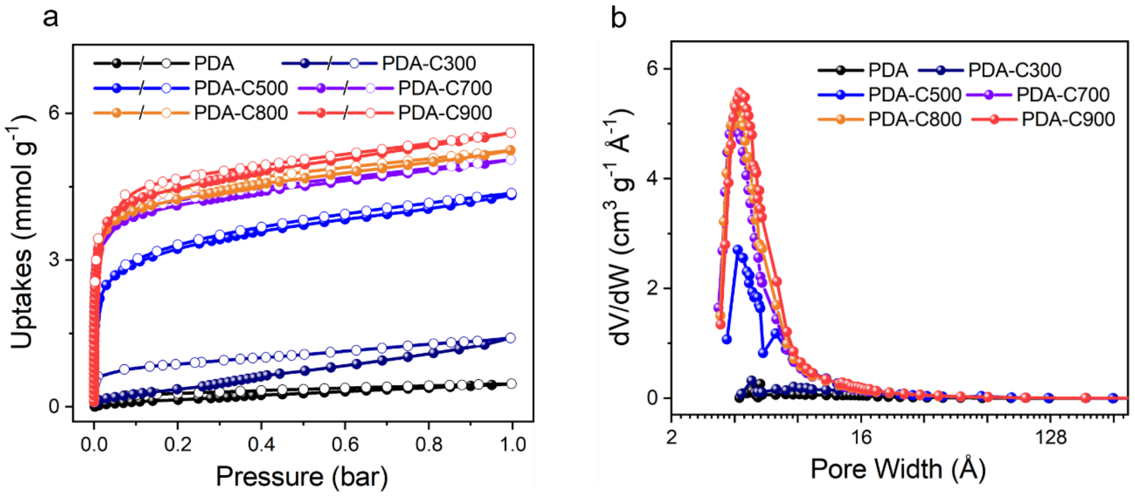

Supplementary Figure 12. (a) CO_2 adsorption (solid circles) and desorption (empty circles) isotherms measured at 195 K for PDA and PDA-Cx and (b) corresponding Horvath-Kawazoe (H-K) pore size distributions.

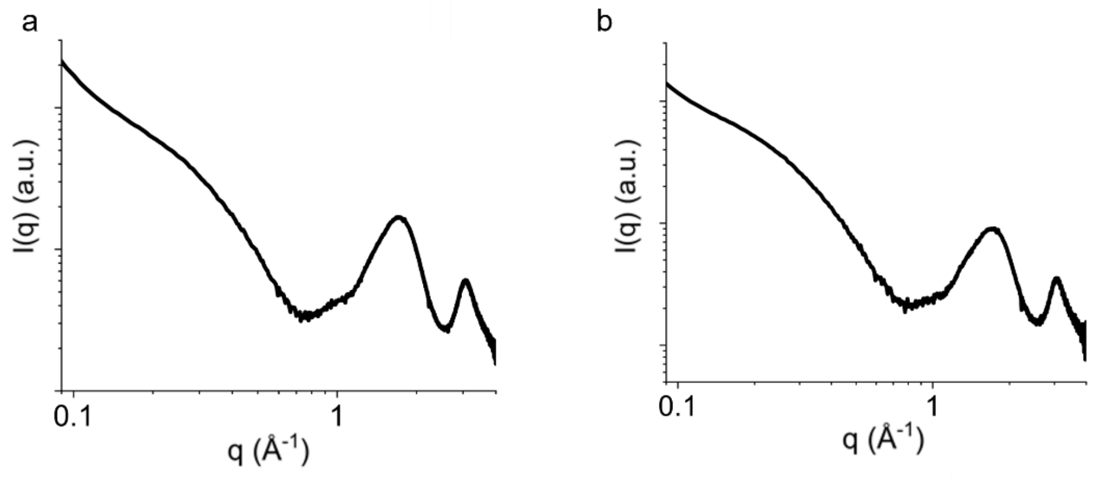

Supplementary Figure 13. The WAXS scattering curve of (a) PDA-C800 and (b) PDA-C900 (The shoulder of scattering at high q values (>0.7 $Å^{-1}$) is a typical characteristic of narrow turbostratic interlayer spacing in carbon matrix. The scattering beyond the shoulder corresponds to the pore walls and atomic disorder.[2])

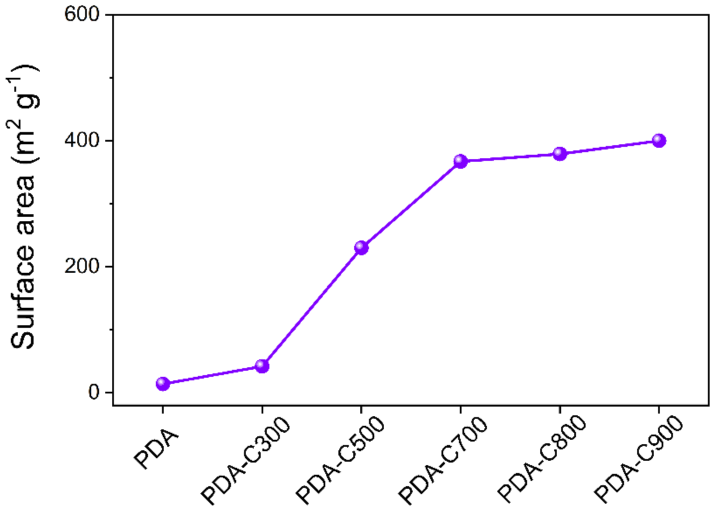

Supplementary Figure 14. Surface area analyzed by CO_2 at 195 K on PDA and PDA-Cx.

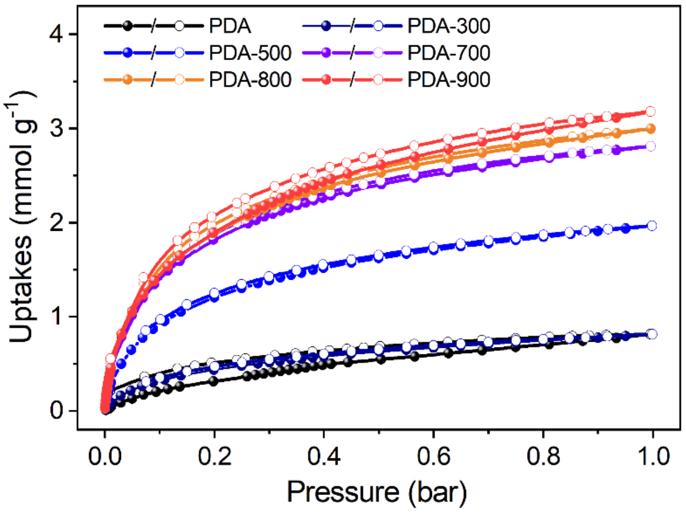

Supplementary Figure 15. (a) CO_2 adsorption (solid circles) and desorption (empty circles) isotherms measured at 273 K for PDA and PDA-Cx.

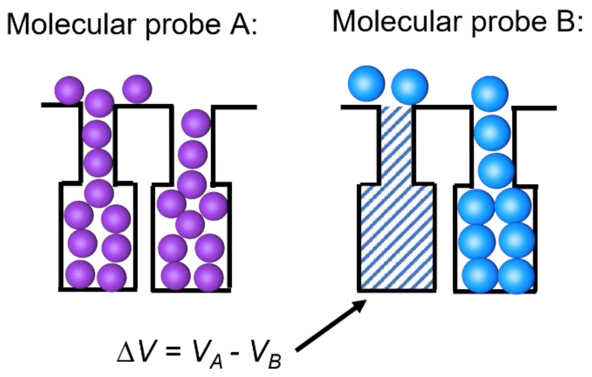

Supplementary Figure 16. Principle of pore accessibility for different probe gases. The difference in calculated pore volume (ΔV) for two probes represents exactly the fraction of its pore system which is sized between both molecules.

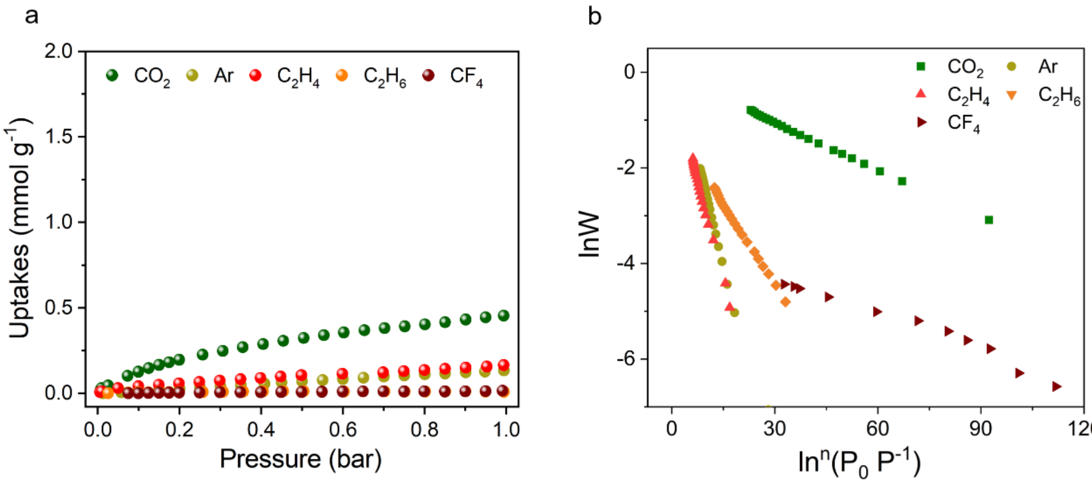

Supplementary Figure 17. a) Probe molecular adsorption isotherms and b) typical D-A plot on PDA-C300 at 273 K.

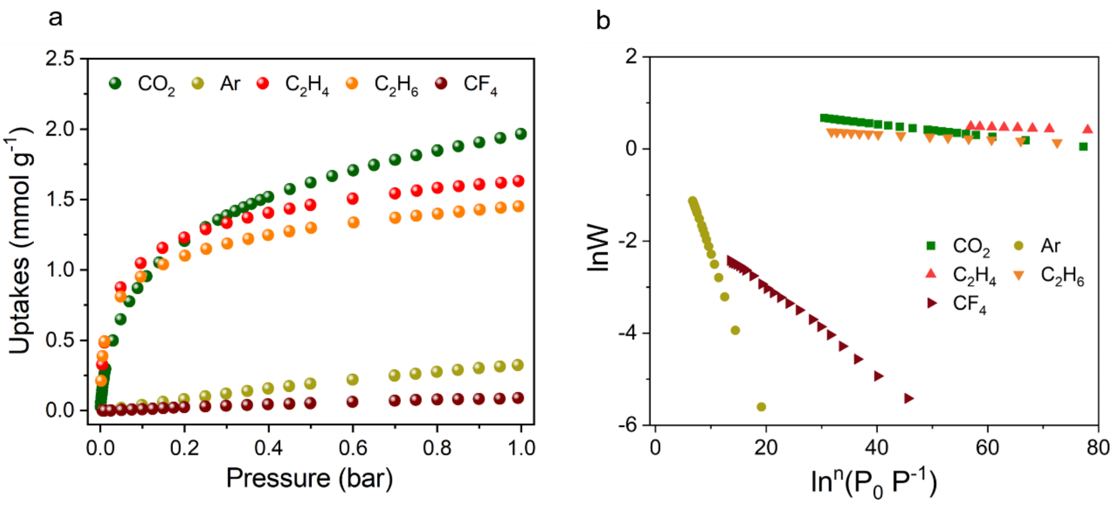

Supplementary Figure 18. a) Probe molecular adsorption isotherms and b) typical D-A plot on PDA-C500 at 273 K.

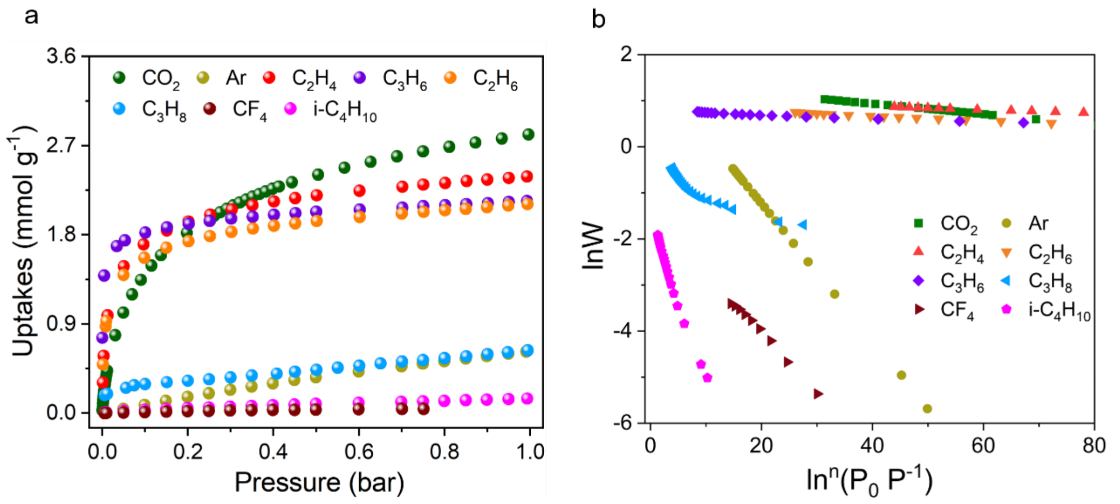

Supplementary Figure 19. a) Probe molecular adsorption isotherms and b) typical D-A plot on PDA-C700 at 273 K.

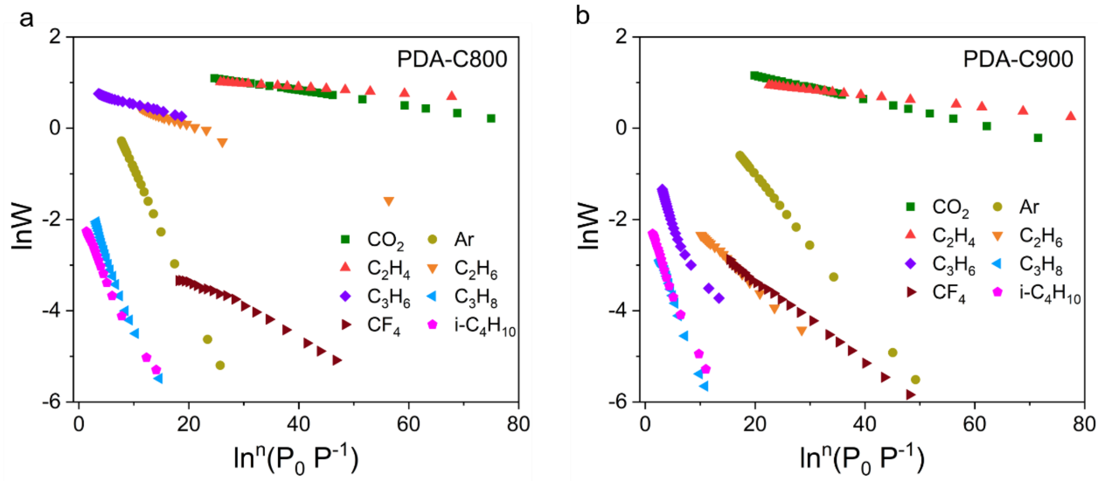

Supplementary Figure 20. Typical D-A plot of probe molecules on (a) PDA-C800 and (b) PDA-C900 at 273 K.

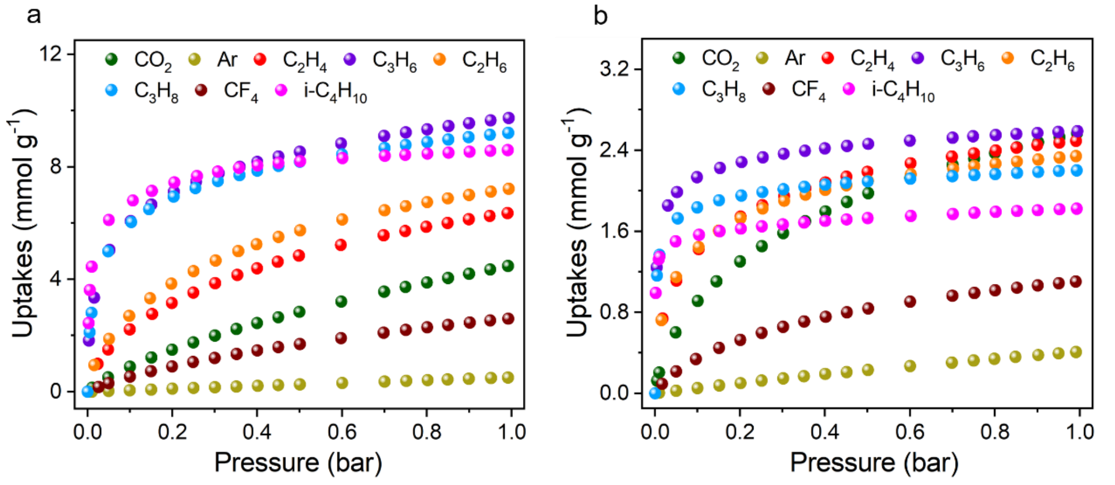

Supplementary Figure 21. Probe molecular adsorption isotherms on (a) AC-1 and (b) CMS-1 at 273 K.

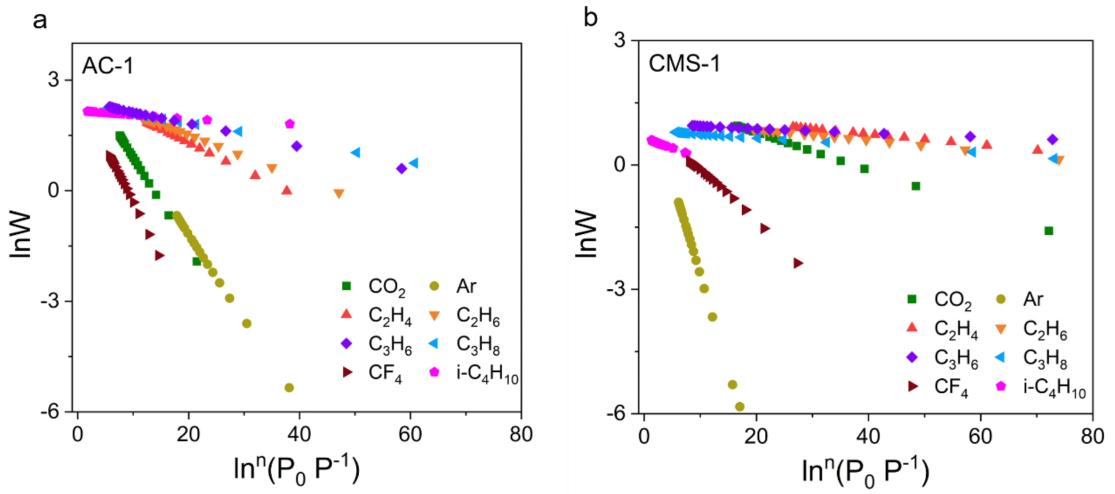

Supplementary Figure 22. Typical D-A plot of probe molecules on (a) AC-1 and (b) CMS-1 at 273 K.

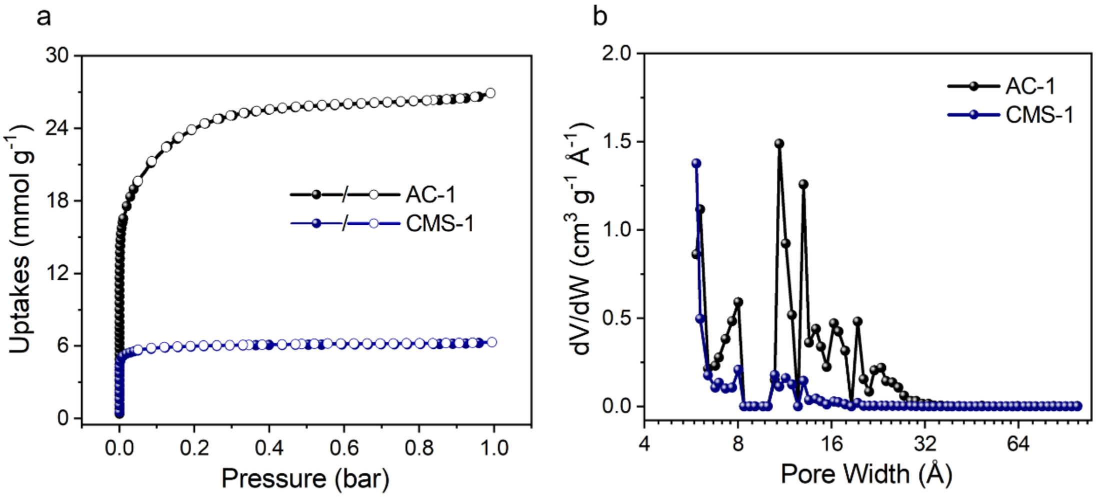

Supplementary Figure 23. (a) N_2 adsorption (solid circles) and desorption (empty circles) isotherms measured at 77 K for AC-1 and CMS-1 samples and (b) corresponding NLDFT pore size distributions.

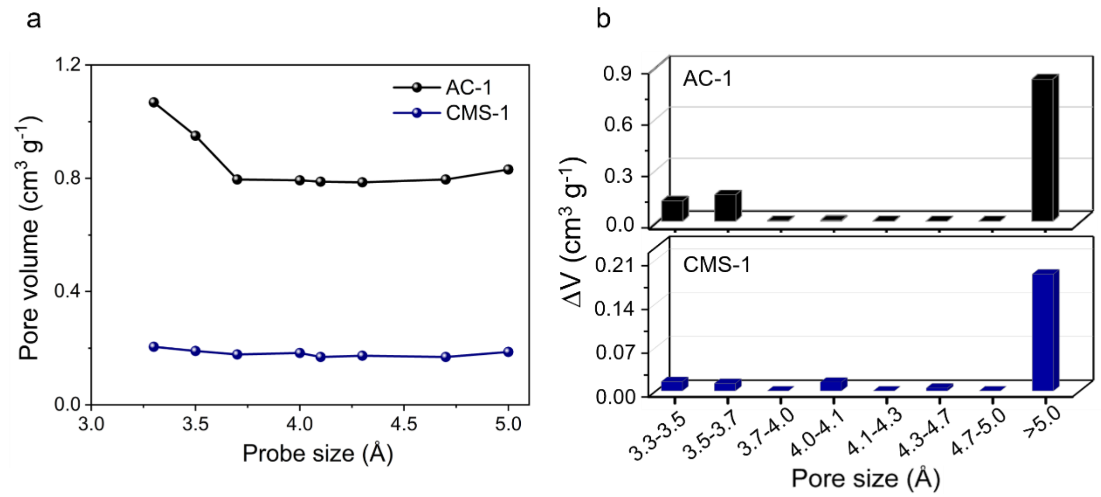

Supplementary Figure 24. a) The pore volumes of AC-1 and CMS-1 calculated from different probe gases based on the Dubinin-Astakhov equation (The data points from left to right are calculated from probing gases of CO_2, Ar, C_2H_4, C_2H_6, C_3H_6, C_3H_8, CF_4, and i-C_4H_{10}); b) The corresponding pore size distributions (The differential pore volume (ΔV) was obtained from probes with successive sizes. V_{Ar} calculated by Ar was subtracted from V_{CO2}, V_{C2H4} from V_{Ar}, and so on. The last one $V_{i\text{-}C4H10}$ was pore volume with a size larger than 5.0 Å).

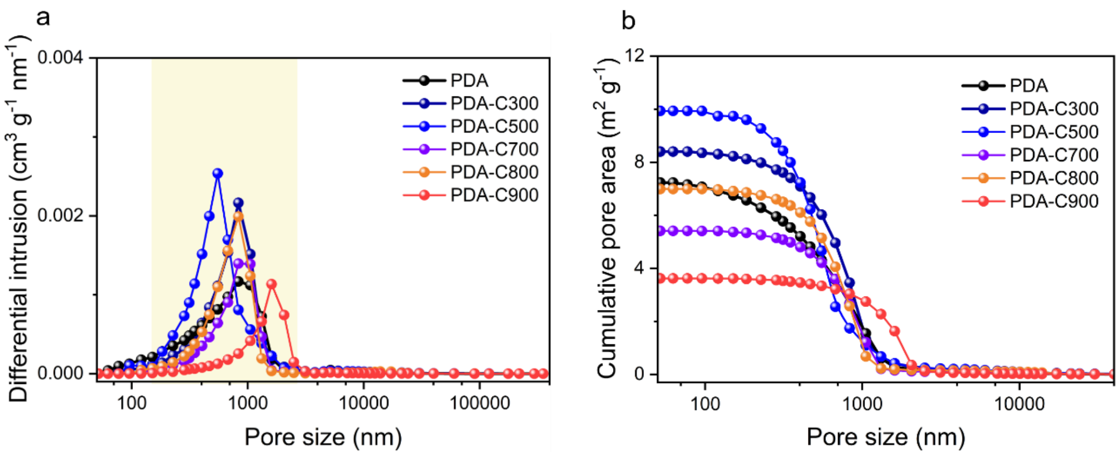

Supplementary Figure 25. Macroporosity fraction of PDA and PDA-Cx obtained from mercury porosimetry.

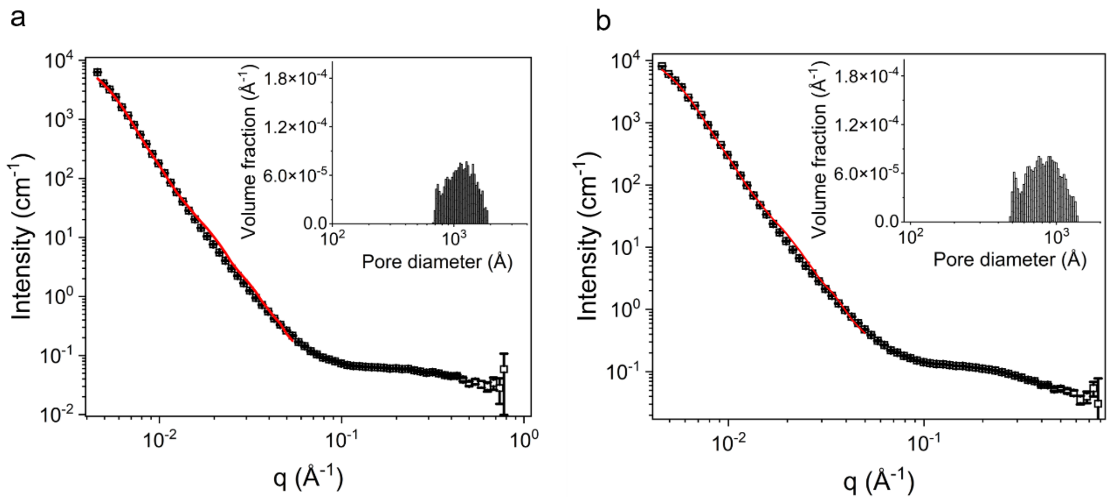

Supplementary Figure 26. SAXS scattering curve on (a) PDA-C800 and (b) PDA-C900 (Insets are the calculated pore size distribution. The corresponding negligible pore volume confirms the absence of obvious mesopores).

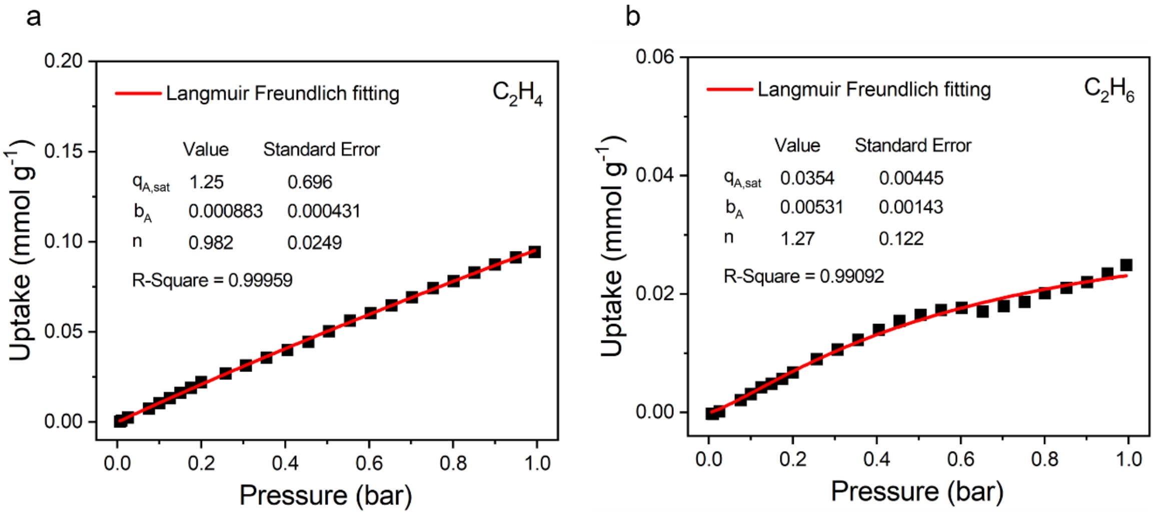

Supplementary Figure 27. C_2H_4 and C_2H_6 sorption data at 298 K for PDA and the corresponding non-linear curve fits.

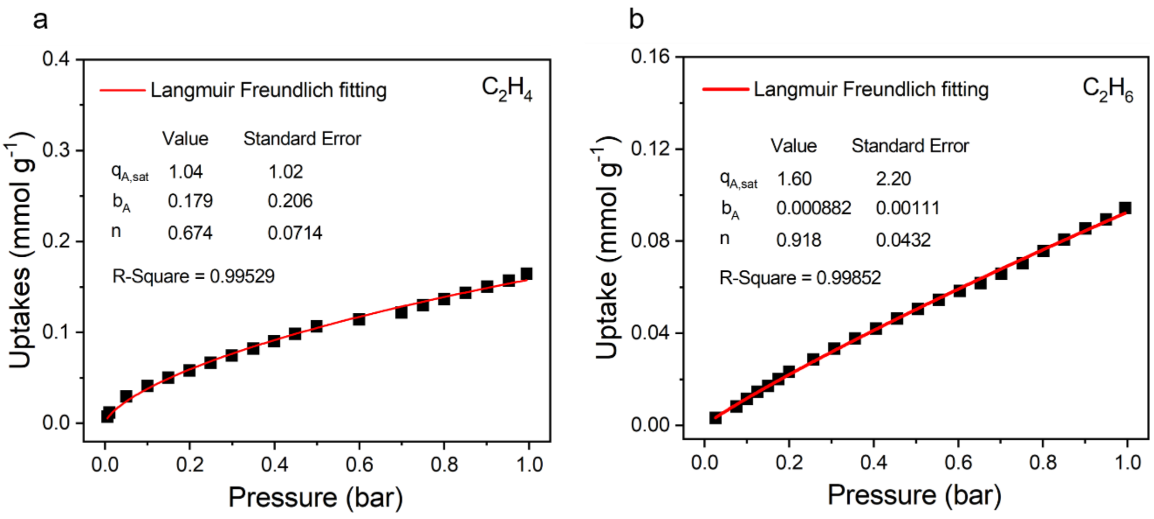

Supplementary Figure 28. C_2H_4 and C_2H_6 sorption data at 298 K for PDA-C300 and the corresponding non-linear curve fits.

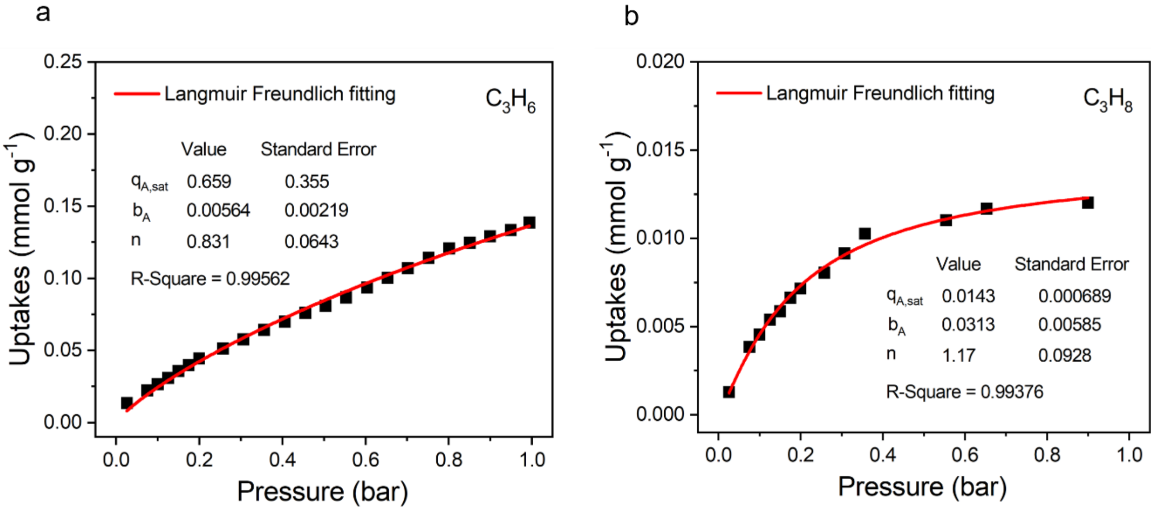

Supplementary Figure 29. C_3H_6 and C_3H_8 sorption data at 298 K for PDA-C300 and the corresponding non-linear curve fits.

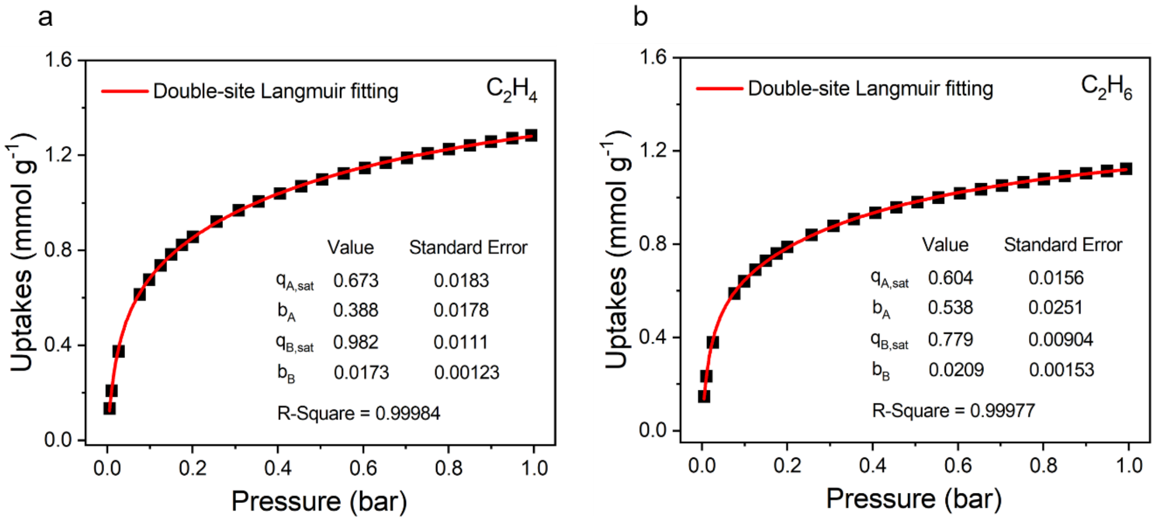

Supplementary Figure 30. C_2H_4 and C_2H_6 sorption data at 298 K for PDA-C500 and the corresponding non-linear curve fits.

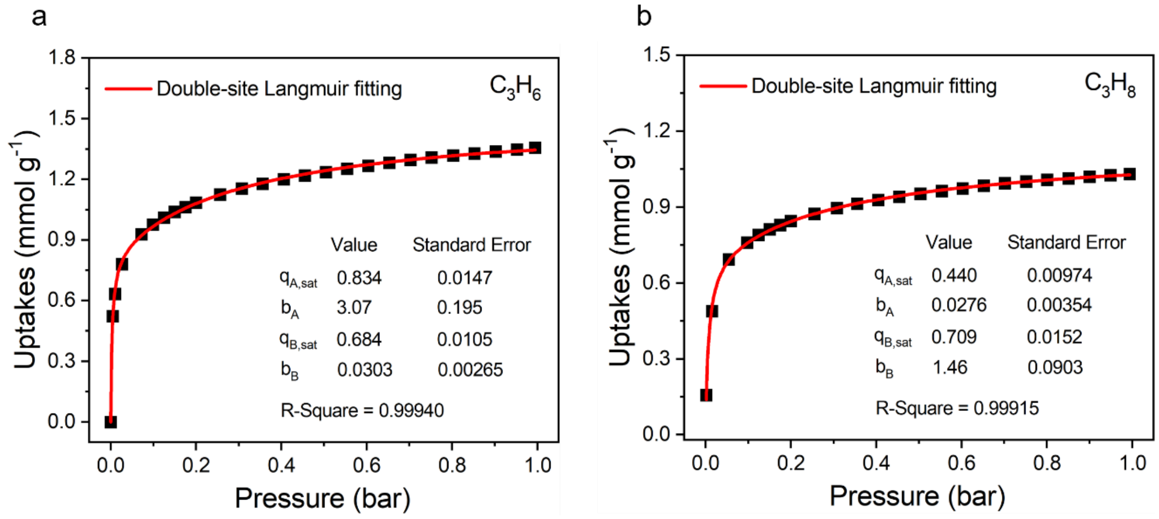

Supplementary Figure 31. C_3H_6 and C_3H_8 sorption data at 298 K for PDA-C500 and the corresponding non-linear curve fits.

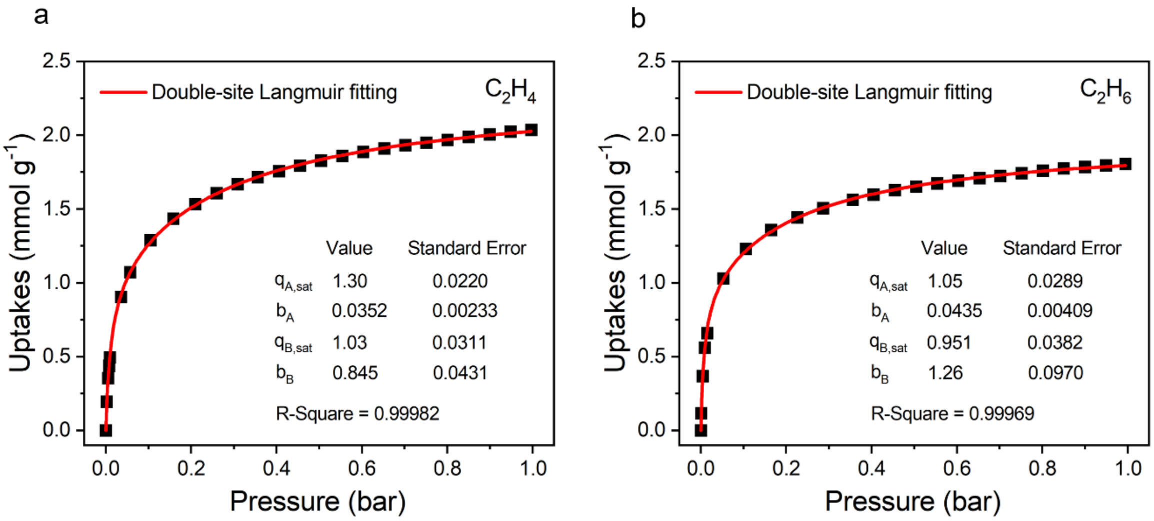

Supplementary Figure 32. C_2H_4 and C_2H_6 sorption data at 298 K for PDA-C700 and the corresponding non-linear curve fits.

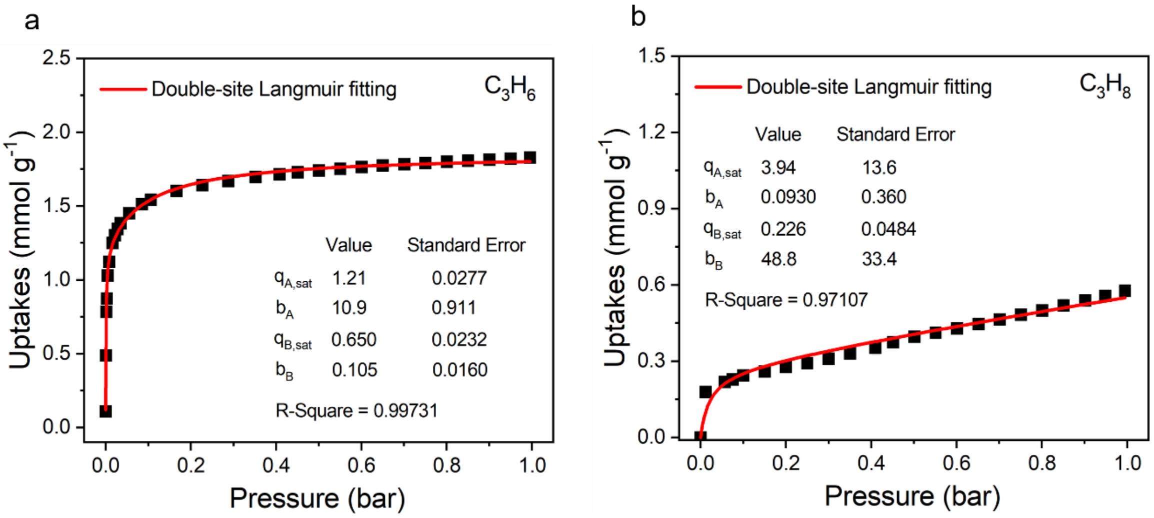

Supplementary Figure 33. C_3H_6 and C_3H_8 sorption data at 298 K for PDA-C700 and the corresponding non-linear curve fits.

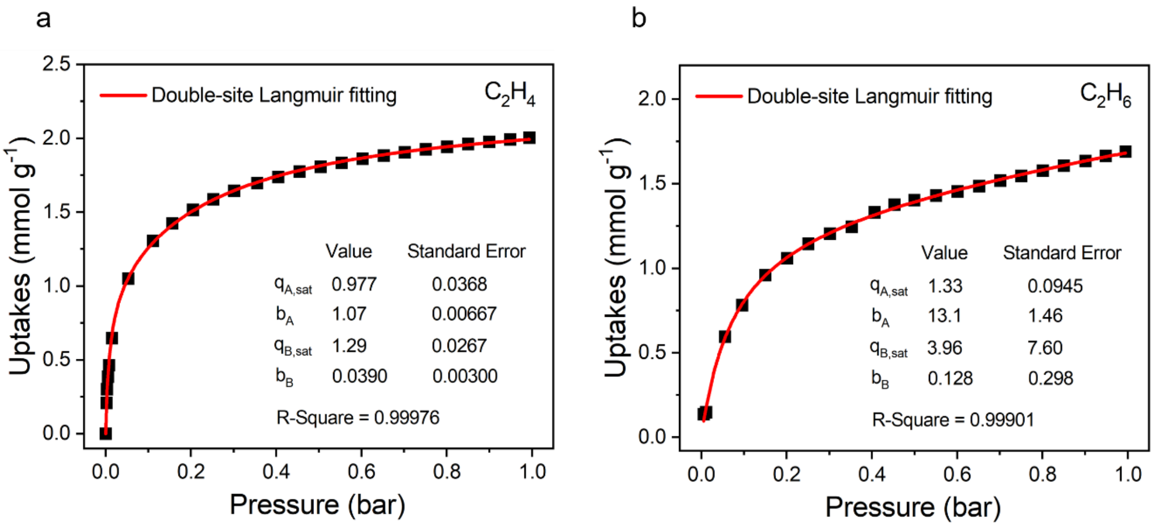

Supplementary Figure 34. C_2H_4 and C_2H_6 sorption data at 298 K for PDA-C800 and the corresponding non-linear curve fits.

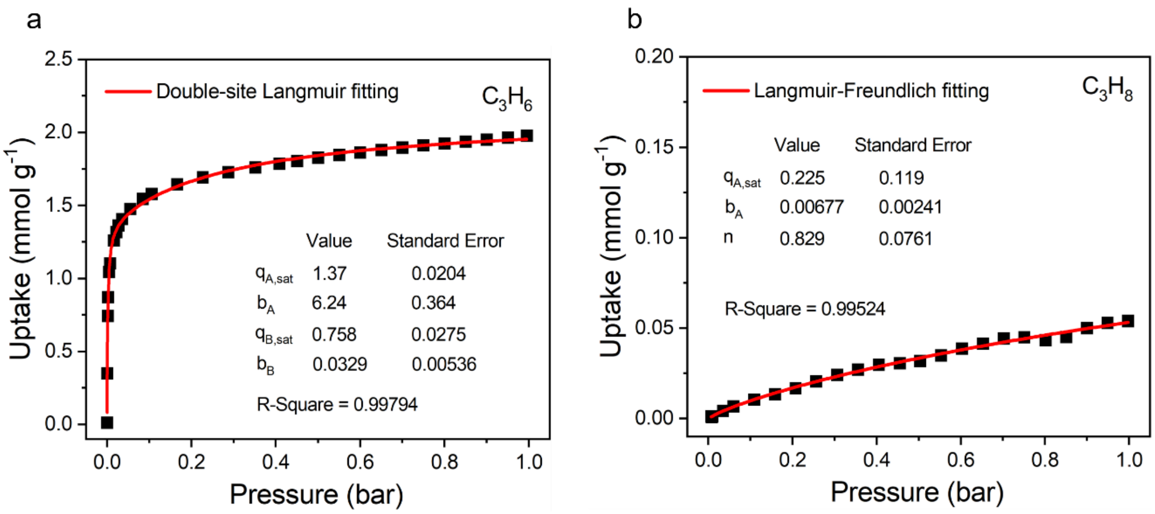

Supplementary Figure 35. C_3H_6 and C_3H_8 sorption data at 298 K for PDA-C800 and the corresponding non-linear curve fits.

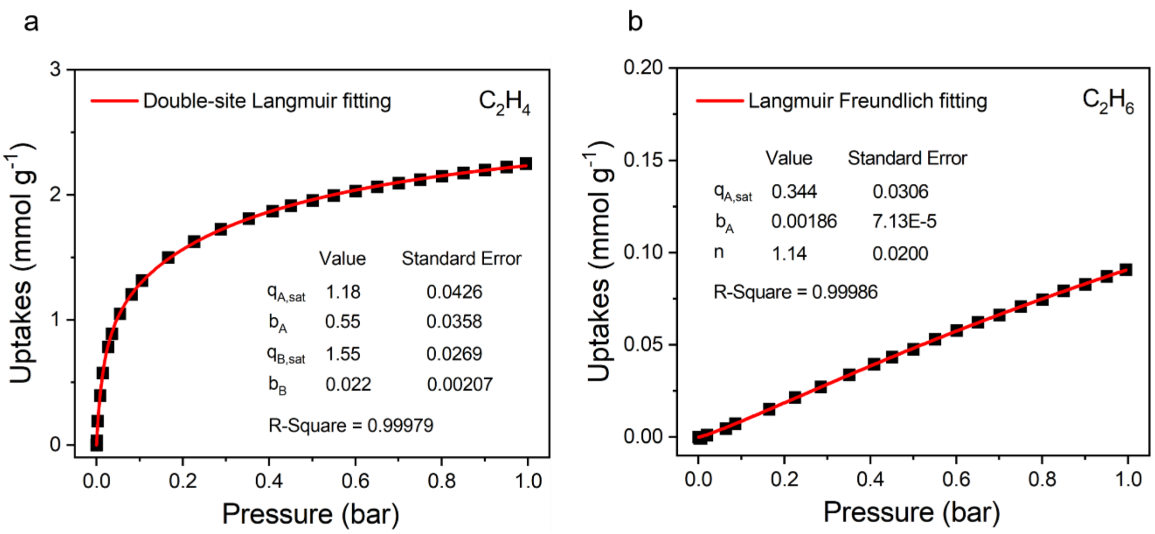

Supplementary Figure 36. C_2H_4 and C_2H_6 sorption data at 298 K for PDA-C900 and the corresponding non-linear curve fits.

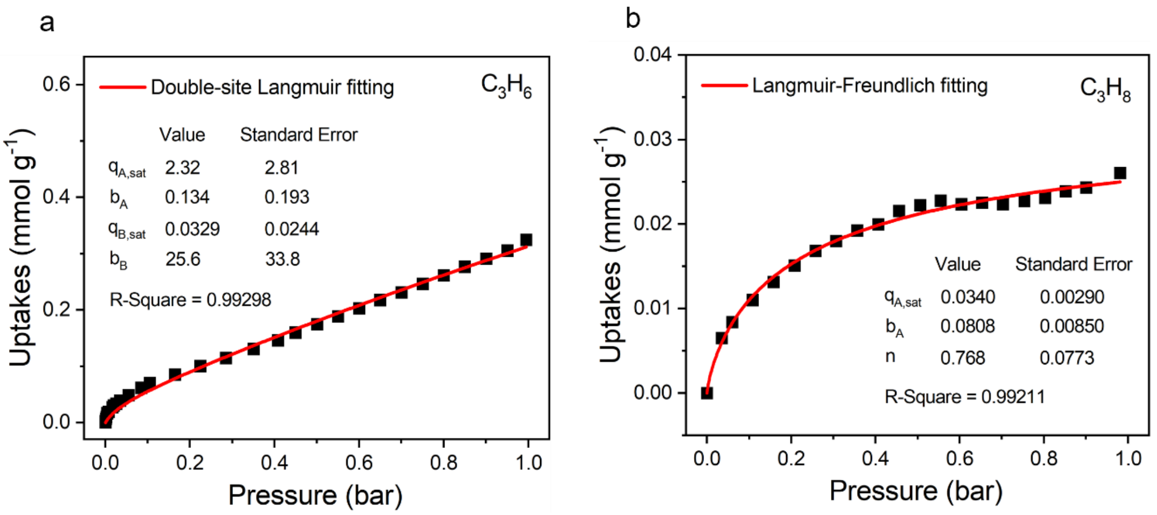

Supplementary Figure 37. C_3H_6 and C_3H_8 sorption data at 298 K for PDA-C900 and the corresponding non-linear curve fits.

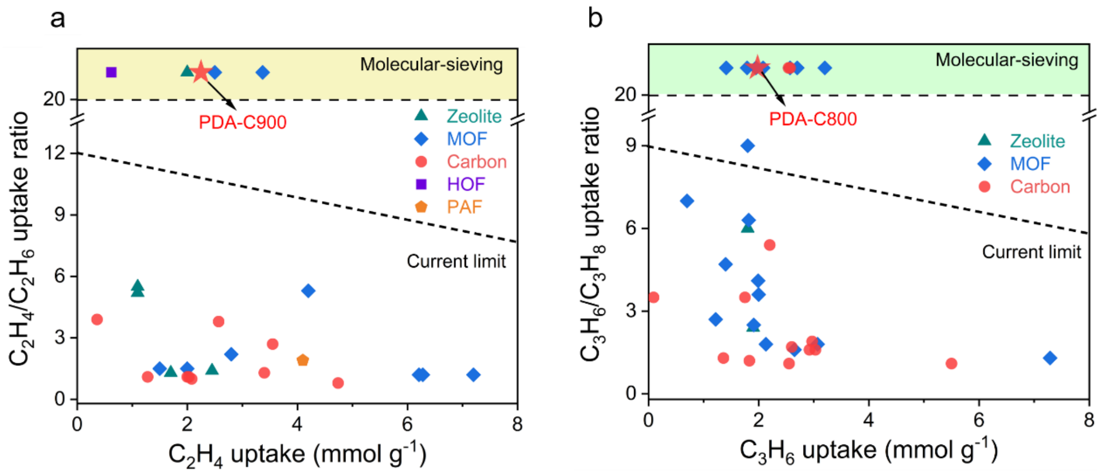

Supplementary Figure 38. Comparison of olefin adsorption capacity and olefin/paraffin separation factor of PDA-C800 and PDA-C900 developed in this work with top-performing adsorbents reported in the literatures (The dotted line is the current limitation of capacity and separation factor. The details are given in Supplementary Tables 7-8. The size-sieving adsorbents are marked in yellow (a) and green (b) regions with olefin/paraffin uptake ratio over 20).

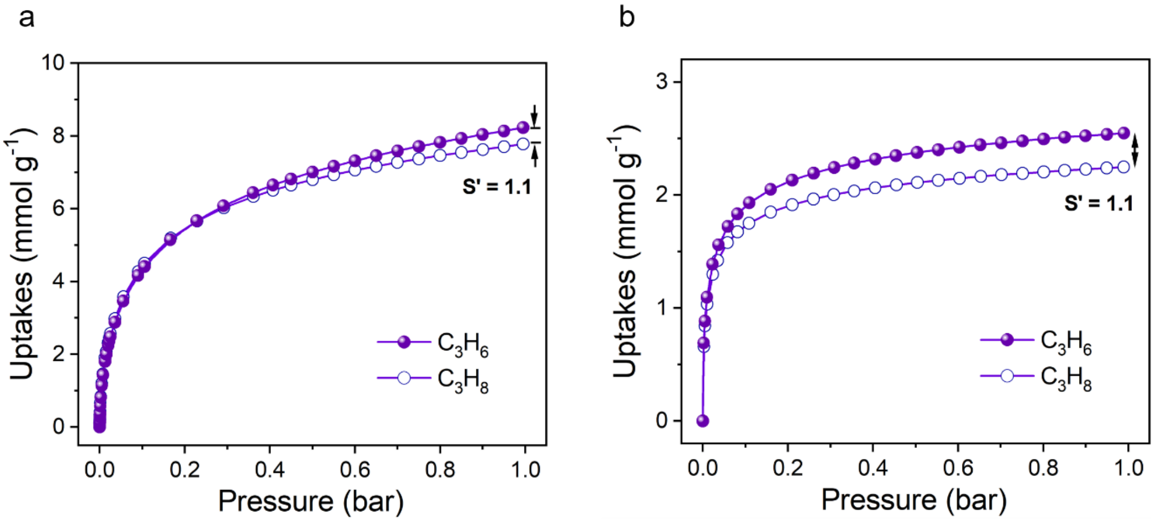

Supplementary Figure 39. C_3H_6 and C_3H_8 sorption isotherms and separation factor of a) AC-1 and b) CMS-1 at 298 K and 0-1.0 bar.

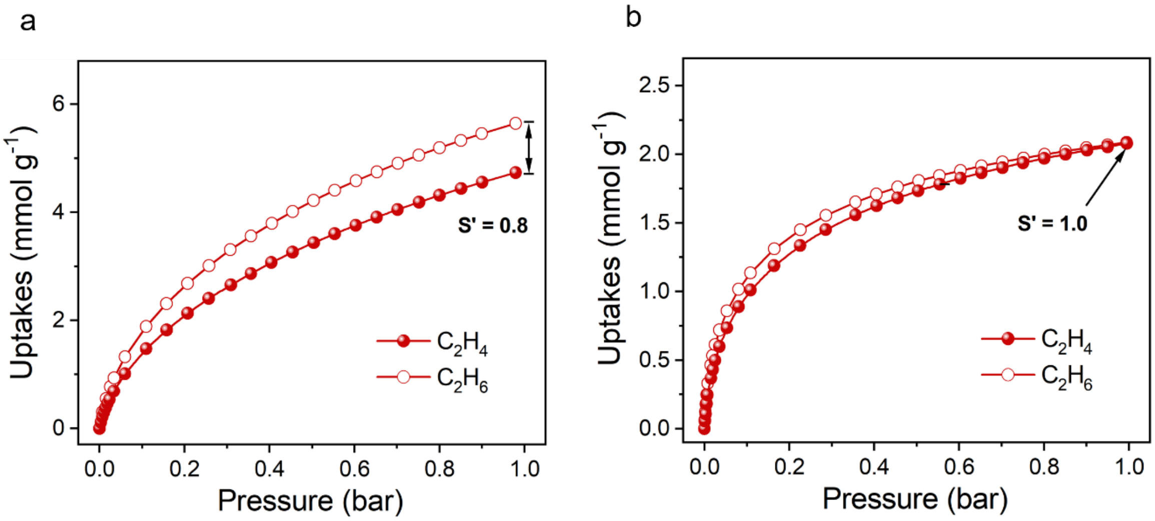

Supplementary Figure 40. C_2H_4 and C_2H_6 sorption isotherms and separation factor of a) AC-1 and b) CMS-1 at 298 K and 0-1.0 bar.

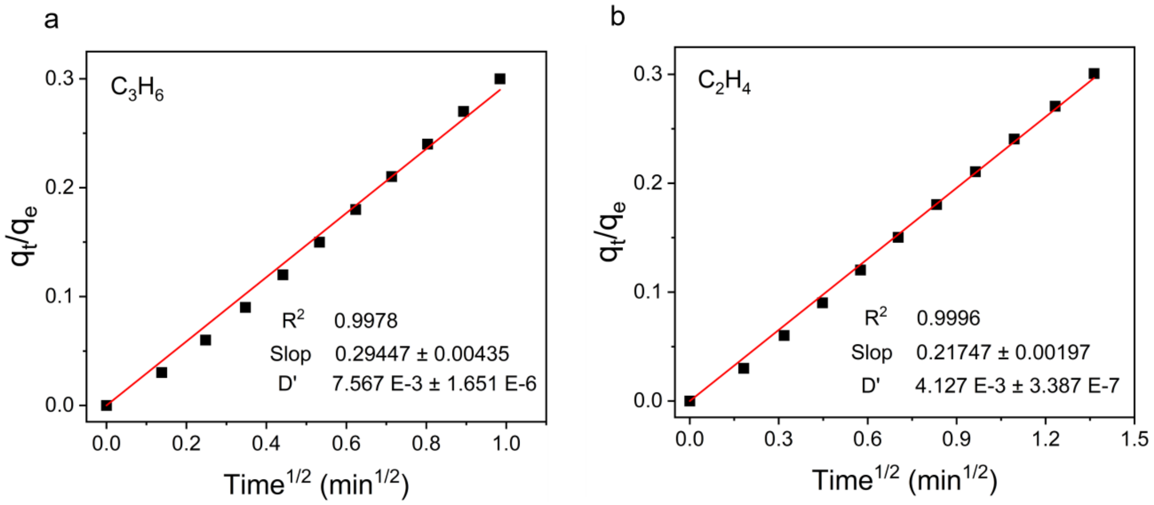

Supplementary Figure 41. The diffusional time constant calculation details for a) C_3H_6 in PDA-C800 and a) C_2H_4 in PDA-C900.

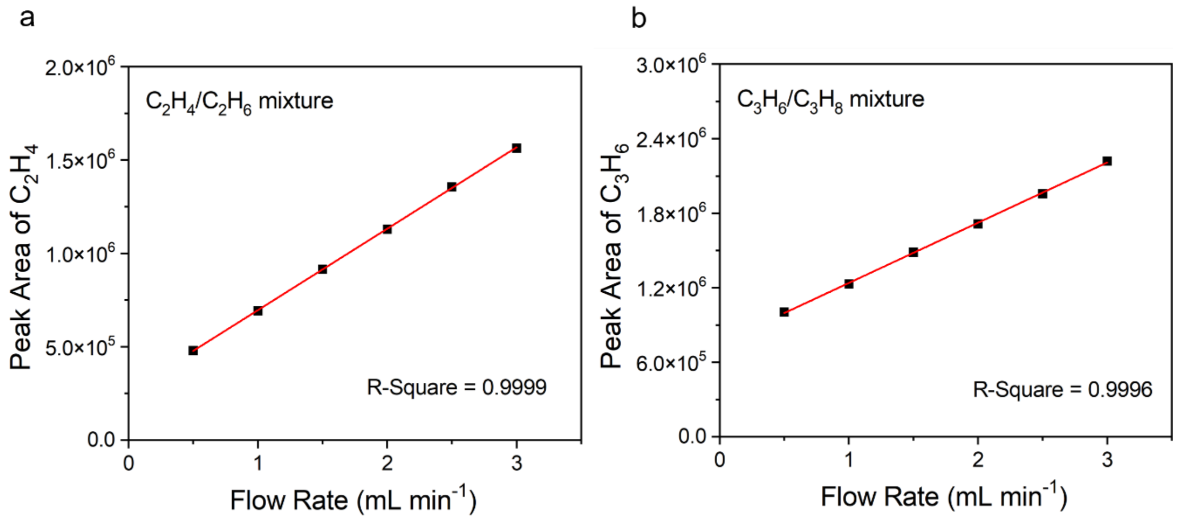

Supplementary Figure 42. Calibration curve of (a) C_2H_4/C_2H_6 gas mixture and (b) C_3H_6/C_3H_8 flow rate versus their peak area of C_2H_4 and C_3H_6 on the gas chromatogram, respectively.

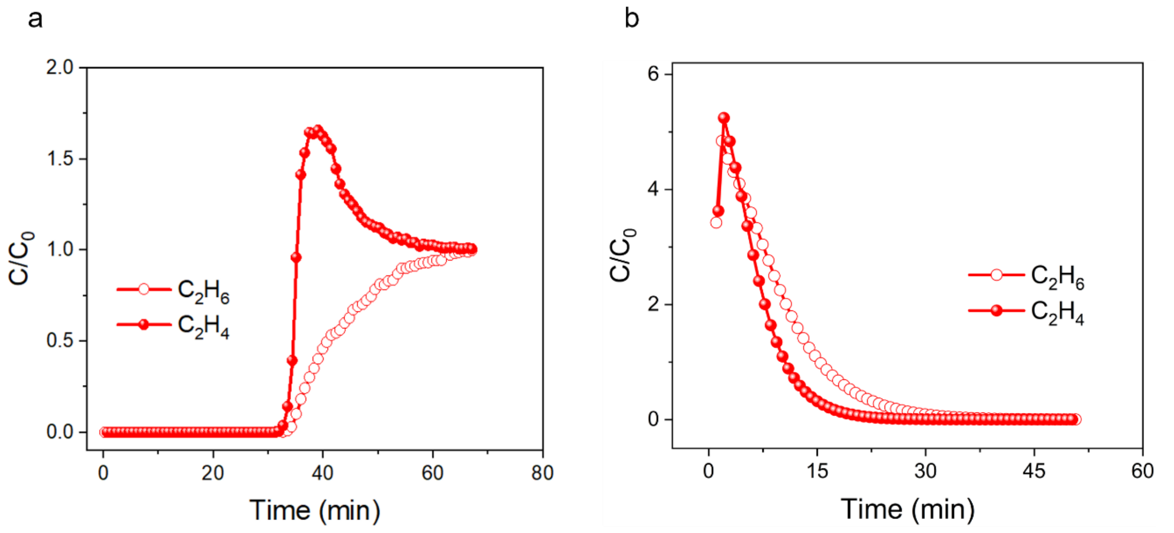

Supplementary Figure 43. (a) Breakthrough curve for an equimolar mixture C_2H_4/C_2H_6 gas mixture on AC-1 sample (dosage of adsorbents: 400 mg); (b) Composition of desorbed gas following a column breakthrough experiment. Desorption was performed by heating the column from room temperature to 353 K under He flow.

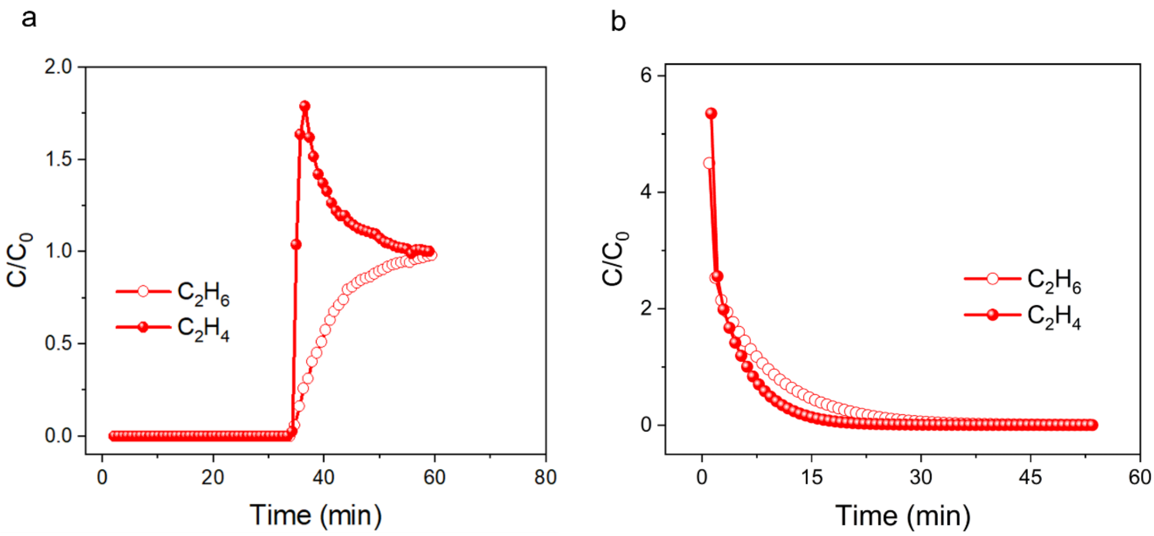

Supplementary Figure 44. (a) Breakthrough curve for an equimolar mixture C_2H_4/C_2H_6 gas mixture on CMS-1 sample (dosage of adsorbents: 1.1 g); (b) Composition of desorbed gas following a column breakthrough experiment. Desorption was performed by heating the column from room temperature to 353 K under He flow.

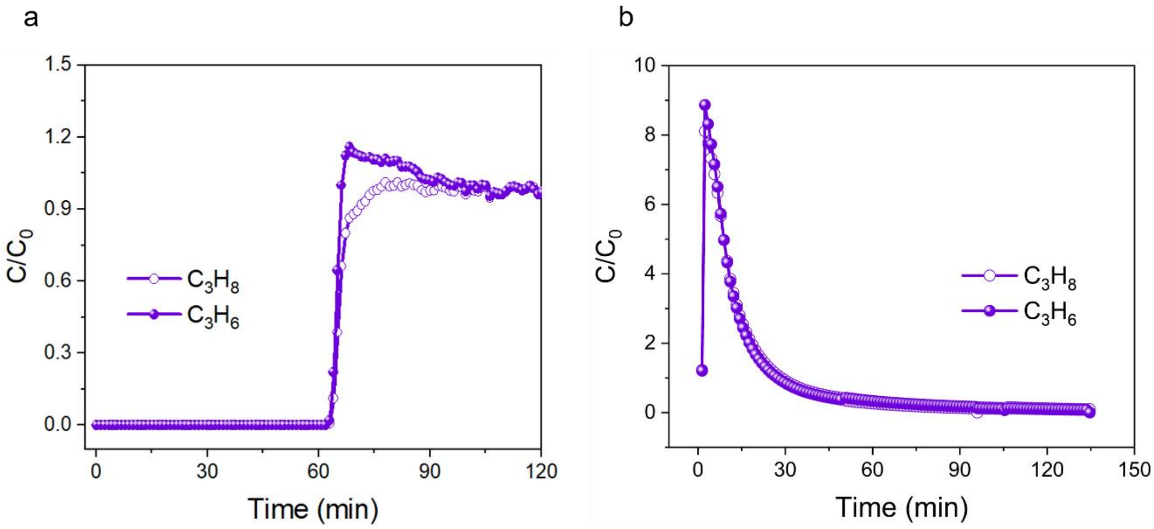

Supplementary Figure 45. (a) Breakthrough curve for an equimolar mixture C_3H_6/C_3H_8 gas mixture on AC-1 sample (dosage of adsorbents: 400 mg); (b) Composition of desorbed gas following a column breakthrough experiment. Desorption was performed by heating the column from room temperature to 353 K under He flow.

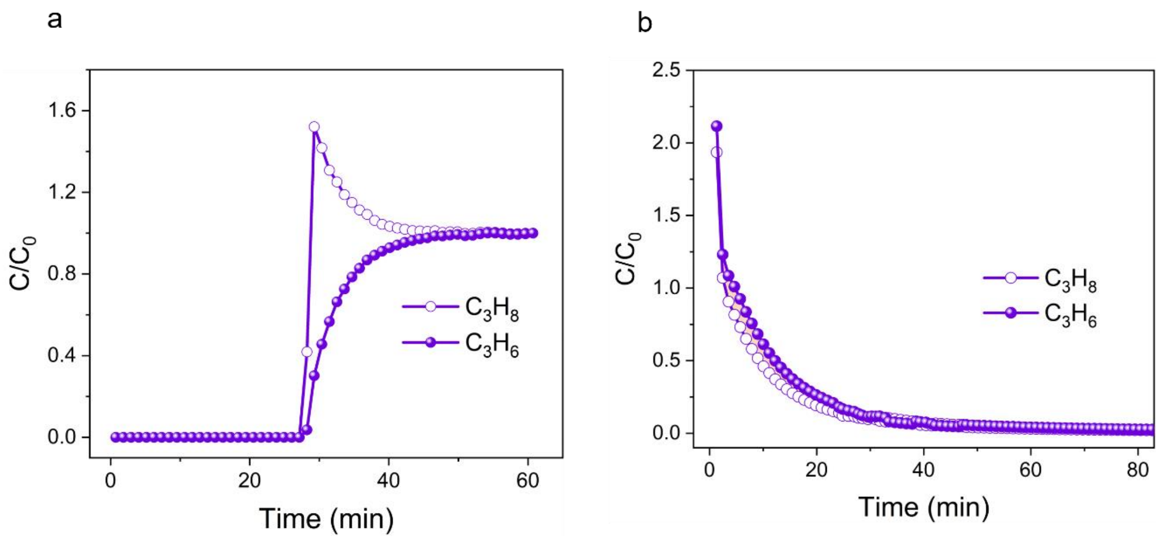

Supplementary Figure 46. (a) Breakthrough curve for an equimolar mixture C_3H_6/C_3H_8 gas mixture on CMS-1 sample (dosage of adsorbents: 1.1 g); (b) Composition of desorbed gas following a column breakthrough experiment. Desorption was performed by heating the column from room temperature to 353 K under He flow.

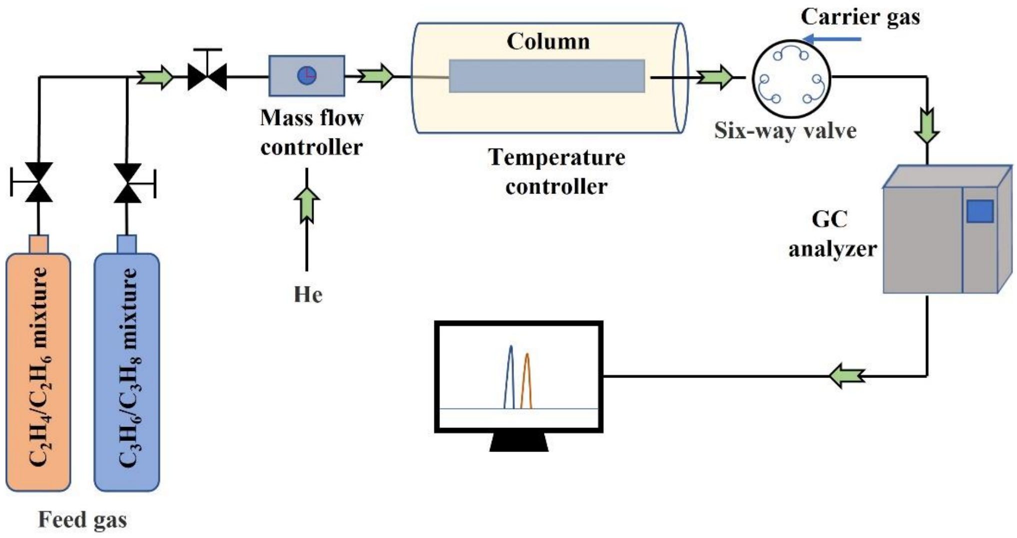

Supplementary Figure 47. Schematic illustration of the apparatus for the breakthrough experiments.

Supplementary Tables. 1-11

Supplementary Table 1. Element Compositions of PDA and PDA-Cx from XPS analysis.

Samples	C%	O%	N%
PDA	71.33	21.77	6.90
PDA-C300	75.88	15.97	8.16
PDA-C500	83.60	8.38	8.02
PDA-C700	87.85	6.02	6.18
PDA-C800	89.04	5.73	5.23
PDA-C900	89.87	6.05	4.09

Supplementary Table 2. Average positron lifetime (τ_1, τ_2, τ_3) and their corresponding intensities of PDA-Cx.

Samples	τ_1 (ps)	I_1 (%)	τ_2 (ps)	I_2 (%)	τ_3 (ns)	I_3 (%)	Fitting variance
PDA-C300	167.3 ± 2.3	18.46 ± 0.14	375.2 ± 0.5	80.73 ± 0.14	1.74 ± 0.14	0.82 ± 0.02	1.0036
PDA-C500	180.7 ± 2.8	18.80 ± 0.42	388.4 ± 0.8	81.12 ± 0.42	5.90 ± 1.70	0.08 ± 0.01	1.0363
PDA-C700	179.9 ± 6.2	18.60 ± 0.91	388.5 ± 2.0	81.10 ± 0.91	2.48 ± 0.20	0.30 ± 0.03	0.9928
PDA-C800	168.7 ± 7.6	12.01 ± 0.56	399.7 ± 1.2	87.99 ± 0.56	--	--	1.0332
PDA-C900	164.8 ± 8.4	11.12 ± 0.45	399.6 ± 1.0	88.88 ± 0.45	--	--	1.0462

Supplementary Table 3. Properties of molecular probes used.

Molecules	Boiling point (K)	Minimum dimension (Å)	Liquid V_{mol} at NBP (cm^3 mol^{-1})	T_c (K)	P_c (bar)	Polarizability $\times 10^{25}$ (cm^3)
CO_2	216.55	3.3	37.4	304.12	73.74	29.11
Ar	87.27	3.5	28.7	150.86	48.98	16.411
C_2H_4	169.42	3.7	49.4	282.34	50.41	42.52
C_2H_6	184.55	4.1	55.0	305.32	48.72	44.3-44.7
C_3H_6	225.46	4.0	69.1	364.90	46.00	62.6
C_3H_8	231.02	4.3	75.7	369.83	42.48	62.9-63.7
CF_4	145.11	4.7	53.5	227.51	37.45	38.38
i-C_4H_{10}	261.34	5.0	97.8	407.85	36.40	81.4-82.9

The dimensions for CO_2, Ar, and CF_4 were adopted from Koros., al.[3] The dimensions of the alkene and alkane molecules were calculated from a DREIDING force field.[4,5]

The calculation of saturated vapor pressure (P_0) of super-critical gases

Under supercritical conditions, the saturation vapor pressure of Ar was used to account for non-ideality.[6] In this work, a modified Dubinin equation given by Amankwah and Schwarz was used to evaluate the virtual saturation vapor pressure (P_0), which is formulated as:[7]

$$P_0 = \mathrm{P_C}\left(\frac{T}{\mathrm{T_C}}\right)^{\mathrm{k}} \tag{4}$$

Here, k is an adjustable parameter to be determined by the specific adsorbent-adsorbate system, P_c and T_c are critical pressure and temperature, respectively.

On the other hands, the adsorbed phase density of super-critical gases is an approximation and cannot be measured directly. The adsorbed phase is assumed to be a superheated liquid with a negligible pressure. Here, the empirical formulas put forward by Ozawa et al.[8] has been used to calculate the adsorbed phase density:

$$\rho_{\mathrm{ad}} = \frac{\rho_{\mathrm{b}}}{\exp\left[0.0025(T-\mathrm{T_b})\right]} \tag{5}$$

Where ρ_{ad} is the adsorbed phase density, T_b is the sorbate boiling point temperature, and ρ_b is the density of the liquid adsorbate at the boiling point.

Supplementary Table 4. Fitting parameters of the D-A equation on PDA-C900, PDA-C800 and PDA-C700.

Gases	PDA-C900			PDA-C800			PDA-C700		
	lnW_0	n	R^2	lnW_0	n	R^2	lnW_0	n	R^2
CO_2	1.70	2.10	0.9998	1.61	2.25	0.9991	1.46	2.42	0.9998
Ar	1.94	1.60	0.9999	1.87	1.46	0.9999	1.72	1.69	0.9999
C_2H_4	1.28	2.16	0.9951	1.26	2.25	0.9961	1.07	2.62	0.9941
C_2H_6	-1.36	1.76	0.9936	0.94	1.83	09967	0.86	2.48	0.9992
C_3H_6	-0.81	1.27	0.9433	0.85	1.44	0.9952	0.76	2.39	0.9932
C_3H_8	-1.93	1.19	0.9958	-1.24	1.41	0.9904	0.46	0.65	0.9536
CF_4	-1.53	2.12	0.9991	-1.88	2.25	0.9902	-1.62	1.80	0.9901
i-C_4H_{10}	-1.96	1.29	0.9912	-1.99	1.42	0.9883	-1.79	1.25	0.9901

Supplementary Table 5. Fitting parameters of the D-A equation on PDA-C500 and PDA-C300.

Gases	PDA-C500			PDA-C300		
	lnW_0	n	R^2	lnW_0	n	R^2
CO_2	1.07	2.40	0.9999	0.33	2.20	0.9999
Ar	1.32	1.37	0.9996	0.55	1.50	0.9900
C_2H_4	0.65	2.80	0.9993	-0.18	1.26	0.9955
C_2H_6	0.50	2.63	0.9985	-1.21	1.90	0.9932
C_3H_6	--	--	--	--	--	--
C_3H_8	--	--	--	--	--	--
CF_4	-1.15	2.02	0.9988	-3.35	2.65	0.9614
i-C_4H_{10}	--	--	--	--	--	--

Supplementary Table 6. Fitting parameters of the D-A equation on AC-1 and CMS-1.

Gases	AC-1			CMS-1		
	$\ln W_0$	n	R^2	$\ln W_0$	n	R^2
CO_2	3.35	1.42	0.9999	1.70	1.97	0.9999
Ar	3.48	1.49	0.9998	1.89	1.27	0.9999
C_2H_4	2.78	1.75	0.9999	1.27	2.27	0.9998
C_2H_6	2.66	1.87	0.9998	1.12	2.26	0.9992
C_3H_6	2.44	1.95	0.9997	0.98	2.41	0.9981
C_3H_8	2.34	2.03	0.9994	0.83	2.12	0.9983
CF_4	2.70	1.35	0.9994	1.16	1.62	0.9971
i-C_4H_{10}	2.14	2.53	0.9911	0.65	1.13	0.9969

Supplementary Table 7. Comparison of adsorption and separation performance of C_2H_4/C_2H_6 on top-performing porous adsorbents at 298 K and 1.0 bar.

	Samples	C_2H_4 uptake (mmol g^{-1})	C_2H_6 uptake (mmol g^{-1})	C_2H_4/C_2H_6 Uptake ratio	Reference
MOF	ITQ-55	1.5[a]	non equil.	--	9
	UTSA-280	2.5	--	>20	10
	Co-gallate	3.37	0.31	10.9	11
	NOTT-300	4.28[b]	0.85[b]	5.3	12
	NUS-36	1.5	1.0	1.5	13
	Fe_2(m-dobdc)	7.2	6.0	1.2	14
	Fe-MOF-74	6.28[c]	5.10[c]	1.2	15
	Co-MOF-74	6.21[c]	5.25[c]	1.2	16
	HKUST-1	7.20[a]	6.03[a]	1.2	17
	MIL-101-SO_3Ag	2.8	1.3	2.2	18
	NUS-6(Hf)-Ag	2.0	1.3	1.5	19
Zeolite	AgA	2	--	>20	20
	Zeolite 5A	2.45[a]	1.72[a]	1.4	21
	Ag–SBA-15	1.1	0.2	5.5	22
	MeOPh-f-MOR	1.1[a]	0.21[a]	5.2	23
	Na-ETS-10	1.7	1.3	1.3	24
PAF[d]	PAF-1-SO3Ag	4.1	2.2	1.9	25
HOF	HOF-FJU-1a	0.62	--	>20	26
Carbon	CuCl(8.0)/AC	2.57	0.67	3.8	27
	8CuCl/CMK-3	3.55	1.33	2.7	28
	MC-S-Ag-3	3.4	2.7	1.3	29
	AC-1	4.74	5.65	0.8	This work
	CMS-1	2.08	2.09	1.0	This work
	PDA-C300	0.36	0.092	3.9	This work
	PDA-C500	1.28	1.12	1.1	This work
	PDA-C700	2.03	1.81	1.1	This work
	PDA-C800	2.00	1.79	1.1	This work
	PDA-C900	2.25	0.091	24.7	This work

[a, b, c] Gas uptake at 303, 293 and 318 K, respectively. [d] Porous aromatic framework.

Supplementary Table 8. Comparison of adsorption and separation performance of C_3H_6/C_3H_8 on top-performing porous adsorbents at 298 K and 1.0 bar.

	Samples	C_3H_6 uptake (mmol g^{-1})	C_3H_8 uptake (mmol g^{-1})	C_3H_6/C_3H_8 Uptake ratio	Reference
MOF	KAUST-7	1.41	--	>20	[30]
	Y-abtc	1.95	--	>20	[31]
	Co-gallate	1.79	0.14	12.8	[32]
	$[Zn_3(OH)_2(pzdc)(atz)]$	2.08	--	>20	[33]
	HIAM-301	3.2	--	>20	[34]
	NJU-Bai8	2.7[a]	--	>20	[35]
	Y-dbai	2.57	0.10	25.7	[36]
	JNU-3a	~1.8[b]	~0.2[b]	9.0	[37]
	CPL-1	1.82[c]	0.29[c]	6.3	[38]
	1to	3.07	1.70	1.8	[39]
	AGTU-3a	1.22	0.46	2.7	[40]
	CAU-10-OMe	1.4[c]	0.3[c]	4.7	[41]
	$Zn_2(5\text{-aip})_2(bpy)$	1.91	0.76	2.5	[42]
	SIFSIX-2-Cu-i	2.65	1.67	1.6	[43]
	$Co_2(dobdc)$	7.29	5.5	1.3	[44]
	$Co(AIP)(BPY)_{0.5}$	1.99	0.48	4.1	[45]
	ZU-36-Ni	2.0	0.55	3.6	[46]
	$ZnAtzPO_4$	2.13	1.19	1.8	[47]
	Milli-Zn-ATA	0.7[d]	0.1[d]	7.0	[48]
Zeolite	Na-A	1.8	0.3	6.0	[49]
	Cs-ZK-5-16-973	1.9	0.8	2.4	[49]
Carbon	SAM-HCP-Ag-3	1.75	0.5	3.5	[50]
	MC-S-Ag-3	5.5	4.8	1.1	[51]
	MC-wiggle	2.6	1.5	1.7	[52]
	CMS-18	2.97	1.59	1.9	[53]
	CNP-3	3.03	1.86	1.6	[54]
	Ag@HCP-AC-1	2.92	1.80	1.6	[55]
	SC-K	2.20	0.41	5.4	[56]
	SCMS-0.2-800	2.54	0.08	31.8	[57]
	C-CDMOF-2-700	1.97	0.13	15.2	[58]
	AC-1	8.23	7.78	1.1	This work
	CMS-1	2.55	2.25	1.1	This work
	PDA-C300	0.14	0.012	11.7	This work
	PDA-C500	1.36	1.03	1.3	This work
	PDA-C700	1.83	0.58	3.2	This work
	PDA-C800	1.98	0.054	36.7	This work
	PDA-C900	0.32	0.026	12.3	This work

[a] Gas uptake at 0.4 bar. [b, c] Gas uptake at 323 and 273 K, respectively. [d] Dynamic gas uptake.

Supplementary Table 9. Peak area of C_2H_4 on gas chromatogram at different flowrates.

C_2H_4 flow rate (mL min^{-1})	Peak area of C_2H_4 on gas chromatogram (5 runs for each flow rate)					
	1	2	3	4	5	Average
0.5	481837	477992	479950	483237	473335	479270
1.0	662671	703795	701433	695624	698584	692421
1.5	911283	914715	916610	915107	920288	915600
2.0	1119808	1136328	1139526	1124662	1131174	1130300
2.5	1345735	1356940	1358310	1364314	1356999	1356460
3.0	1568582	1569681	1565422	1551253	1557780	1562544

Supplementary Table 10. Peak area of C_3H_6 on gas chromatogram at different flowrates.

C_3H_6 flow rate (mL min^{-1})	Peak area of C_3H_6 on gas chromatogram (5 runs for each flow rate)					
	1	2	3	4	5	Average
0.5	1011530	998737	1010200	1003489	995506	1003892
1.0	1236603	1216017	1243622	1243254	1216420	1231183
1.5	1478331	1470126	1489008	1502700	1492586	1486550
2.0	1693599	1718488	1730033	1713232	1713232	1713717
2.5	1956074	1958538	1944469	1957571	1971267	1957584
3.0	2177574	2234740	2250368	2233902	2202097	2219736

Supplementary Table 11. Fitting parameters of the virial equation and the corresponding correlation coefficients.

	a_0	a_1	a_2	a_3	a_4	R^2
C_2H_4	-3890.26	16.6033	0.28916	-0.00445	2.52E-05	0.9985
C_3H_6	-2981.62	-35.158	0.26814	0.00863	-7.28E-5	0.9973

Supplementary References

1. Li, L. et al. Ethane/ethylene separation in a metal-organic framework with iron-peroxo sites. *Science* **362**, 443-446 (2018).
2. László, K., Czakkel, O., Josepovits, K., Rochas, C. & Geissler, E. Influence of Surface Chemistry on the SAXS Response of Polymer-Based Activated Carbons. *Langmuir* **21**, 8443-8451 (2005).
3. Rungta, M., Xu, L. & Koros, W.J. Structure–performance characterization for carbon molecular sieve membranes using molecular scale gas probes. *Carbon* **85**, 429-442 (2015).
4. Mayo, S.L., Olafson, B.D. & Goddard, W.A. DREIDING: a generic force field for molecular simulations. *Journal of Physical chemistry* **94**, 8897-8909 (1990).
5. Hayashi, J.-i. et al. Separation of ethane/ethylene and propane/propylene systems with a carbonized BPDA− pp ʻODA polyimide membrane. *Industrial & engineering chemistry research* **35**, 4176-4181 (1996).
6. Wu, S., Tang, D., Li, S., Chen, H. & Wu, H. Coalbed methane adsorption behavior and its energy variation features under supercritical pressure and temperature conditions. *Journal of Petroleum Science and Engineering* **146**, 726-734 (2016).
7. Amankwah, K. & Schwarz, J. A modified approach for estimating pseudo-vapor pressures in the application of the Dubinin-Astakhov equation. *Carbon* **33**, 1313-1319 (1995).
8. Ozawa, S., Kusumi, S. & Ogino, Y. Physical adsorption of gases at high pressure. IV. An improvement of the Dubinin—Astakhov adsorption equation. *Journal of Colloid and Interface Science* **56**, 83-91 (1976).
9. Bereciartua, P.J. et al. Control of zeolite framework flexibility and pore topology for separation of ethane and ethylene. *Science* **358**, 1068-1071 (2017).
10. Lin, R.-B. et al. Molecular sieving of ethylene from ethane using a rigid metal–organic framework. *Nature Materials* **17**, 1128-1133 (2018).
11. Bao, Z. et al. Molecular Sieving of Ethane from Ethylene through the Molecular Cross-Section Size Differentiation in Gallate-based Metal–Organic Frameworks. *Angewandte Chemie* **130**, 16252-16257 (2018).
12. Yang, S. et al. Supramolecular binding and separation of hydrocarbons within a functionalized porous metal–organic framework. *Nature Chemistry* **7**, 121-129 (2015).
13. Wang, Y. et al. Pore Size Reduction in Zirconium Metal–Organic Frameworks for Ethylene/Ethane Separation. *ACS Sustainable Chemistry & Engineering* **7**, 7118-7126 (2019).
14. Bachman, J.E., Kapelewski, M.T., Reed, D.A., Gonzalez, M.I. & Long, J.R. M2(m-dobdc) (M = Mn, Fe, Co, Ni) Metal–Organic Frameworks as Highly Selective, High-Capacity Adsorbents for Olefin/Paraffin Separations. *Journal of the American Chemical Society* **139**, 15363-15370 (2017).

15. Bloch, E.D. et al. Hydrocarbon Separations in a Metal-Organic Framework with Open Iron(II) Coordination Sites. *Science* **335**, 1606-1610 (2012).
16. Geier, S.J. et al. Selective adsorption of ethylene over ethane and propylene over propane in the metal–organic frameworks M2(dobdc) (M = Mg, Mn, Fe, Co, Ni, Zn). *Chemical Science* **4**, 2054-2061 (2013).
17. He, Y., Krishna, R. & Chen, B. Metal–organic frameworks with potential for energy-efficient adsorptive separation of light hydrocarbons. *Energy & Environmental Science* **5**, 9107-9120 (2012).
18. Zhang, Y. et al. Highly selective adsorption of ethylene over ethane in a MOF featuring the combination of open metal site and π-complexation. *Chemical communications* **51**, 2714-2717 (2015).
19. Wang, Y., Hu, Z., Cheng, Y. & Zhao, D. Silver-decorated hafnium metal–organic framework for ethylene/ethane separation. *Industrial & Engineering Chemistry Research* **56**, 4508-4516 (2017).
20. Aguado, S., Bergeret, G., Daniel, C. & Farrusseng, D. Absolute Molecular Sieve Separation of Ethylene/Ethane Mixtures with Silver Zeolite A. *Journal of the American Chemical Society* **134**, 14635-14637 (2012).
21. Mofarahi, M. & Salehi, S.M. Pure and binary adsorption isotherms of ethylene and ethane on zeolite 5A. *Adsorption* **19**, 101-110 (2013).
22. Wu, C. et al. Comparative Study of Ag+-Based Adsorbents Performance in Ethylene/Ethane Separation. *Journal of Chemical & Engineering Data* **64**, 611-618 (2019).
23. Lee, H.S. et al. Post-Synthesis Functionalization Enables Fine-Tuning the Molecular-Sieving Properties of Zeolites for Light Olefin/Paraffin Separations. *Advanced Materials* **33**, 2105398 (2021).
24. Anson, A., Wang, Y., Lin, C., Kuznicki, T. & Kuznicki, S. Adsorption of ethane and ethylene on modified ETS-10. *Chemical Engineering Science* **63**, 4171-4175 (2008).
25. Li, B. et al. Introduction of π-Complexation into Porous Aromatic Framework for Highly Selective Adsorption of Ethylene over Ethane. *Journal of the American Chemical Society* **136**, 8654-8660 (2014).
26. Yang, Y. et al. Ethylene/ethane separation in a stable hydrogen-bonded organic framework through a gating mechanism. *Nature Chemistry* **13**, 933-939 (2021).
27. Gao, F., Wang, Y., Wang, X. & Wang, S. Ethylene/ethane separation by CuCl/AC adsorbent prepared using CuCl2 as a precursor. *Adsorption-Journal of the International Adsorption Society* **22**, 1013-1022 (2016).
28. Jiang, W.-J., Sun, L.-B., Yin, Y., Song, X.-L. & Liu, X.-Q. Ordered Mesoporous Carbon CMK-3 Modified with Cu(I) for Selective Ethylene/Ethane Adsorption. *Separation Science and Technology* **48**, 968-976 (2013).
29. Saha, D. et al. Separation of ethane-ethylene and propane-propylene by Ag(I) doped and sulfurized microporous carbon. *Microporous and Mesoporous Materials* **299**, 110099 (2020).
30. Cadiau, A., Adil, K., Bhatt, P.M., Belmabkhout, Y. & Eddaoudi, M. A metal-organic framework-based splitter for separating propylene from propane. *Science* **353**, 137-140 (2016).
31. Wang, H. et al. Tailor-made microporous metal–organic frameworks for the full separation of propane from propylene through selective size exclusion. *Advanced materials* **30**, 1805088 (2018).
32. Liang, B. et al. An ultramicroporous metal–organic framework for high sieving separation of propylene from propane. *Journal of the American Chemical Society* **142**, 17795-17801 (2020).
33. Zhang, X.-W., Zhou, D.-D. & Zhang, J.-P. Tuning the gating energy barrier of metal-organic framework for molecular sieving. *Chem* **7**, 1006-1019 (2021).

34. Yu, L. et al. Pore Distortion in a Metal-Organic Framework for Regulated Separation of Propane and Propylene. *Journal of the American Chemical Society* **143**, 19300-19305 (2021).
35. Wang, X. et al. Guest-dependent pressure induced gate-opening effect enables effective separation of propene and propane in a flexible MOF. *Chemical Engineering Journal* **346**, 489-496 (2018).
36. Tu, S. et al. A new yttrium-based metal–organic framework for molecular sieving of propane from propylene with high propylene capacity. *AIChE Journal* **68**, e17551 (2022).
37. Zeng, H. et al. Orthogonal-array dynamic molecular sieving of propylene/propane mixtures. *Nature* **595**, 542-548 (2021).
38. Chen, Y. et al. Efficient adsorptive separation of C3H6 over C3H8 on flexible and thermoresponsive CPL-1. *Chemical Engineering Journal* **328**, 360-367 (2017).
39. Hu, P. et al. Customized H-bonding acceptor and aperture chemistry within a metal-organic framework for efficient C3H6/C3H8 separation. *Chemical Engineering Journal* **426**, 131302 (2021).
40. Chang, Z., Lin, R.-B., Ye, Y., Duan, C. & Chen, B. Construction of a thiourea-based metal-organic framework with open Ag+ sites for the separation of propene/propane mixtures. *Journal of Materials Chemistry A* **7**, 25567-25572 (2019).
41. Tan, Q. et al. A temperature-responsive smart molecular gate in a metal–organic framework for task-specific gas separation. *Journal of Materials Chemistry A* **7**, 26574-26579 (2019).
42. Chen, Y. et al. A pillar-layer metal-organic framework for efficient adsorption separation of propylene over propane. *Separation and Purification Technology* **204**, 75-80 (2018).
43. Wang, X. et al. Efficient Separation of Propene and Propane Using Anion-Pillared Metal-Organic Frameworks. *Industrial & Engineering Chemistry Research* **59**, 3531-3537 (2020).
44. Bae, Y.-S. et al. High Propene/Propane Selectivity in Isostructural Metal–Organic Frameworks with High Densities of Open Metal Sites. *Angewandte Chemie International Edition* **51**, 1857-1860 (2012).
45. Wu, H. et al. Efficient adsorptive separation of propene over propane through a pillar-layer cobalt-based metal–organic framework. *AIChE Journal* **66**, e16858 (2020).
46. Zhang, Z., Ding, Q., Cui, X., Jiang, X.-M. & Xing, H. Fine-Tuning and Selective-Binding within an Anion-Functionalized Ultramicroporous Metal–Organic Framework for Efficient Olefin/Paraffin Separation. *ACS Applied Materials & Interfaces* **12**, 40229-40235 (2020).
47. Ding, Q. et al. Separation of propylene and propane with a microporous metal–organic framework via equilibrium-kinetic synergetic effect. *AIChE Journal* **67**, e17094 (2021).
48. Ding, Q. et al. Control of intracrystalline diffusion in a bilayered metal-organic framework for efficient kinetic separation of propylene from propane. *Chemical Engineering Journal* **434**, 134784 (2022).
49. Gi Min, J., Christian Kemp, K., Kencana, K.S., Mukti, R.R. & Bong Hong, S. Dealuminated Cs-ZK-5 zeolite for propylene/propane separation. *Chemical Engineering Journal* **413**, 127422 (2021).
50. Stephenson, A. et al. Efficient separation of propane and propene by a hypercrosslinked polymer doped with Ag(i). *Journal of Materials Chemistry A* **7**, 25521-25525 (2019).
51. Saha, D. et al. Separation of ethane-ethylene and propane-propylene by Ag(I) doped and sulfurized microporous carbon. *Microporous and Mesoporous Materials* **299**(2020).
52. Yuan, Y.-F. et al. Wiggling Mesopores Kinetically Amplify the Adsorptive Separation of Propylene/Propane. *Angewandte Chemie-International Edition* **60**, 19063-19067 (2021).
53. Liu, J. et al. New carbon molecular sieves for propylene/propane separation with high working capacity and separation factor. *Carbon* **123**, 273-282 (2017).

54. Xu, S. et al. Beyond the Selectivity-Capacity Trade-Off: Ultrathin Carbon Nanoplates with Easily Accessible Ultramicropores for High-Efficiency Propylene/Propane Separation. *Nano Letters* **22**, 6615-6621 (2022).
55. Xiong, Y. et al. Understanding trade-offs in adsorption capacity, selectivity and kinetics for propylene/propane separation using composites of activated carbon and hypercrosslinked polymer. *Chemical Engineering Journal* **426**, 131628 (2021).
56. Du, S. et al. Ultramicroporous carbons featuring sub-Ångstrom tunable apertures for the selective separation of light hydrocarbon. *AIChE Journal* **67**, e17285 (2021).
57. Du, S., Huang, J., Anjum, A.W., Xiao, J. & Li, Z. A novel mechanism of controlling ultramicropore size in carbons at sub-angstrom level for molecular sieving of propylene/propane mixtures. *Journal of Materials Chemistry A* **9**, 23873-23881 (2021).
58. Chen, F. et al. Molecular Sieving of Propylene from Propane in Metal–Organic Framework-Derived Ultramicroporous Carbon Adsorbents. *ACS Applied Materials & Interfaces* **14**, 30443-30453 (2022).
