# Supplementary material for: Probing sub-5 Ångstrom micropores in carbon for precise light olefin/paraffin separation
Source: Nat Commun. 2023 Mar 2;14:1197. doi: 10.1038/s41467-023-36890-6 (PMC9981619; doi:10.1038/s41467-023-36890-6)
Supplement: Supplementary file 3 — Reporting Summary [file 41467_2023_36890_MOESM3_ESM.pdf]

nature portfolio

Corresponding author(s): Banglin Chen

Last updated by author(s): Feb 9, 2023

Reporting Summary

Nature Portfolio wishes to improve the reproducibility of the work that we publish. This form provides structure for consistency and transparency in reporting. For further information on Nature Portfolio policies, see our Editorial Policies and the Editorial Policy Checklist.

Statistics

For all statistical analyses, confirm that the following items are present in the figure legend, table legend, main text, or Methods section.

n/a	Confirmed	
☐	☒	The exact sample size (n) for each experimental group/condition, given as a discrete number and unit of measurement
☐	☒	A statement on whether measurements were taken from distinct samples or whether the same sample was measured repeatedly
☒	☐	The statistical test(s) used AND whether they are one- or two-sided<br>*Only common tests should be described solely by name; describe more complex techniques in the Methods section.*
☒	☐	A description of all covariates tested
☒	☐	A description of any assumptions or corrections, such as tests of normality and adjustment for multiple comparisons
☒	☐	A full description of the statistical parameters including central tendency (e.g. means) or other basic estimates (e.g. regression coefficient) AND variation (e.g. standard deviation) or associated estimates of uncertainty (e.g. confidence intervals)
☒	☐	For null hypothesis testing, the test statistic (e.g. F, t, r) with confidence intervals, effect sizes, degrees of freedom and P value noted<br>*Give P values as exact values whenever suitable.*
☒	☐	For Bayesian analysis, information on the choice of priors and Markov chain Monte Carlo settings
☒	☐	For hierarchical and complex designs, identification of the appropriate level for tests and full reporting of outcomes
☒	☐	Estimates of effect sizes (e.g. Cohen's d, Pearson's r), indicating how they were calculated

Our web collection on statistics for biologists contains articles on many of the points above.

Software and code

Policy information about availability of computer code

Data collection	No software is used.
Data analysis	We have not used code in this study.

For manuscripts utilizing custom algorithms or software that are central to the research but not yet described in published literature, software must be made available to editors and reviewers. We strongly encourage code deposition in a community repository (e.g. GitHub). See the Nature Portfolio guidelines for submitting code & software for further information.

Data

Policy information about availability of data

All manuscripts must include a data availability statement. This statement should provide the following information, where applicable:

- Accession codes, unique identifiers, or web links for publicly available datasets
- A description of any restrictions on data availability
- For clinical datasets or third party data, please ensure that the statement adheres to our policy

The authors declare that all data supporting the conclusions of this work are available in the article and its Supplementary Information. The further source data will be made available on the request from the corresponding authors.

Human research participants

Policy information about studies involving human research participants and Sex and Gender in Research.

Reporting on sex and gender	This information has not been collected.
Population characteristics	This information has not been collected.
Recruitment	This information has not been collected.
Ethics oversight	This information has not been collected.

Note that full information on the approval of the study protocol must also be provided in the manuscript.

Field-specific reporting

Please select the one below that is the best fit for your research. If you are not sure, read the appropriate sections before making your selection.

☐ Life sciences ☐ Behavioural & social sciences ☒ Ecological, evolutionary & environmental sciences

For a reference copy of the document with all sections, see nature.com/documents/nr-reporting-summary-flat.pdf

Ecological, evolutionary & environmental sciences study design

All studies must disclose on these points even when the disclosure is negative.

Study description	Our study experimentally synthesized the porous materials, probed the narrowed small micropores, and verified their efficiency in precisely size-sieving separation of olefins/paraffins.
Research sample	This is not relevant to our study, because our research focused on probing sub-5 Å micropores in carbon for gas separation.
Sampling strategy	No sample-size calculation was performed. The experimental measurements for adsorption performance of adsorbents were repeated more than three times to ensure data validation.
Data collection	The data in this study were collected by analytical instruments with fixed parameters that were described in the submitted manuscript. The data were recorded by S.-J.D using the software of OriginPro 2022, the first author of this manuscript.
Timing and spatial scale	The data in this study were collected from March 2021 to October 2022. The experiments were done with the cooperation of labs in the mentioned affiliations.
Data exclusions	No data were excluded from the analyses.
Reproducibility	We synthesized the main adsorbents in this study more than five time within one and a half years, and confirmed the consistence of adsorption separation performance. All attempts to repeat the experiment are successful.
Randomization	This is not relevant to our study, because our research does not involve sample allocation.
Blinding	Blinding was not relevant to our study

Did the study involve field work? ☐ Yes ☒ No

Reporting for specific materials, systems and methods

We require information from authors about some types of materials, experimental systems and methods used in many studies. Here, indicate whether each material, system or method listed is relevant to your study. If you are not sure if a list item applies to your research, read the appropriate section before selecting a response.

Materials & experimental systems

n/a	Involved in the study
☒	☐ Antibodies
☒	☐ Eukaryotic cell lines
☒	☐ Palaeontology and archaeology
☒	☐ Animals and other organisms
☒	☐ Clinical data
☒	☐ Dual use research of concern

Methods

n/a	Involved in the study
☒	☐ ChIP-seq
☒	☐ Flow cytometry
☒	☐ MRI-based neuroimaging
